# Reproducible and fully automated testing of nocifensive behavior in mice

**DOI:** 10.1016/j.crmeth.2023.100650

**Published:** 2023-11-21

**Authors:** Christopher Dedek, Mehdi A. Azadgoleh, Steven A. Prescott

**Affiliations:** 1 Neurosciences and Mental Health, The Hospital for Sick Children, Toronto, ON M5G 0A4, Canada; 2 Institute of Biomedical Engineering, University of Toronto, Toronto, ON M5S 3G9, Canada; 3 Department of Physiology, University of Toronto, Toronto, ON M5S 1A8, Canada

**Keywords:** pain, nociception, somatosensory, behavior, optogenetics, sensitivity, reproducibility, automation, artificial intelligence, machine learning

## Abstract

Pain in rodents is often inferred from their withdrawal from noxious stimulation. Threshold stimulus intensity or response latency is used to quantify pain sensitivity. This usually involves applying stimuli by hand and measuring responses by eye, which limits reproducibility and throughput. We describe a device that standardizes and automates pain testing by providing computer-controlled aiming, stimulation, and response measurement. Optogenetic and thermal stimuli are applied using blue and infrared light, respectively. Precise mechanical stimulation is also demonstrated. Reflectance of red light is used to measure paw withdrawal with millisecond precision. We show that consistent stimulus delivery is crucial for resolving stimulus-dependent variations in withdrawal and for testing with sustained stimuli. Moreover, substage video reveals “spontaneous” behaviors for consideration alongside withdrawal metrics to better assess the pain experience. The entire process was automated using machine learning. RAMalgo (reproducible automated multimodal algometry) improves the standardization, comprehensiveness, and throughput of preclinical pain testing.

## Highlights

• Poor reproducibility has plagued preclinical pain research and demands improvement
• We present a technology to standardize stimulation and precisely measure withdrawal
• Machine learning automates aiming and quantification of evoked and non-evoked behaviors
• Automation improves reproducibility, throughput, and comprehensiveness of pain testing

## Motivation

Preclinical pain testing suffers from poor reproducibility, low throughput, and a restricted focus on reflex responses. To address the first two problems, we sought to replace manual stimulation and response measurement with a pain-testing robot. To address the third problem, the robot collects video amenable to the latest machine-learning-based analysis tools to quantify non-reflexive behaviors. Last, to facilitate creation of large datasets, we developed software to store and organize collected data.

## Graphical abstract

**Figure:**
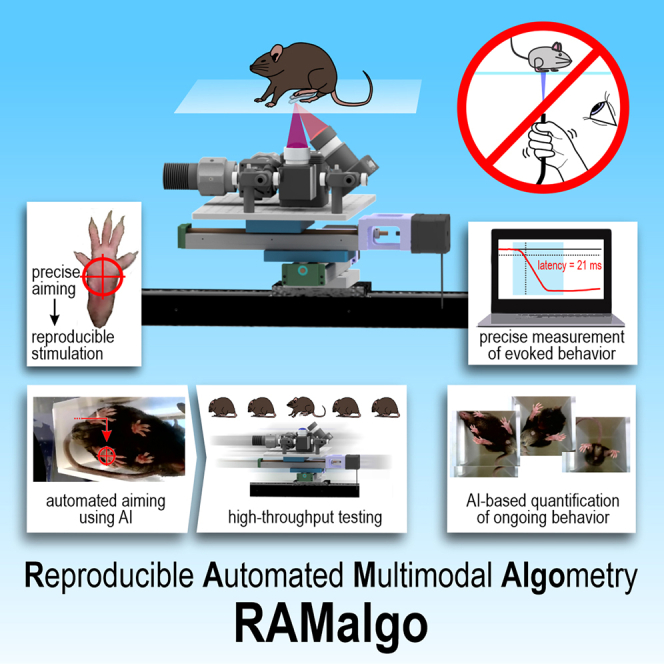

## Teaser

Preclinical pain testing traditionally utilizes techniques—such as stimulating by hand and measuring responses by eye—that limit reproducibility. Dedek et al. describe a device for consistent stimulation and withdrawal measurement with millisecond precision. Testing can be fully automated, increasing standardization and throughput while objectively quantifying non-evoked behaviors.

## Introduction

Measuring withdrawal from noxious stimuli in laboratory rodents is a mainstay of preclinical pain research.^1^^,^^2^^,^^3^^,^^4^ Testing is often conducted on the hind paw, in part because many chronic pain models are designed to increase paw sensitivity through manipulations of the paw or the nerves innervating it.^4^^,^^5^^,^^6^^,^^7^ Measuring evoked pain with withdrawal reflexes has been criticized^8^ because ongoing (non-evoked) pain is a bigger clinical problem,^9^ but tactile and thermal sensitivity are altered in many chronic pain conditions,^10^ and allodynia and spontaneous pain tend to be correlated in human studies^11^^,^^12^ and in some^13^ but not all^14^ mouse studies. Furthermore, sensory profiling is useful for stratifying patients in clinical trials,^15^^,^^16^ and altered sensitivity is often diagnostic.^17^ Ongoing pain should be assessed in addition to, not instead of, evoked pain.^18^ But the most problematic aspects of this testing must be rectified; for instance, outcomes of the hot water tail flick test depend more on who conducts the testing than on any other factor.^19^ This has received scant attention compared with other factors, such as sex.^20^ Outdated technology and poorly standardized testing protocols contribute to the oft-cited reproducibility crisis^21^ and are overdue for transformative improvements.

Preclinical pain tests typically measure withdrawal threshold using brief repeated (incrementing) stimuli such as von Frey filaments or sustained stimuli such as radiant heat. The stimulus intensity (force or skin temperature) at which withdrawal occurs is assumed to be the lowest intensity perceived as painful (i.e., pain threshold), notwithstanding certain caveats.^22^ Withdrawal might not always be triggered by pain, and focusing on threshold fails to consider pain intensity over a broader stimulus range. Recent studies have quantified responses to suprathreshold mechanical stimulation using high-speed video,^23^^,^^24^^,^^25^ but despite precise response measurement, stimuli were delivered by hand, and throughput was low. Resolving subtle changes in pain sensitivity requires that stimulus-response relationships be measured with high resolution (which requires reproducible stimulation and precise response measurement), over a broad dynamic range, and with reasonable efficiency (throughput). Improvements in one factor may come at the expense of other factors. The best compromise depends on the particular experiment, but improving reproducibility and throughput would be a huge benefit.

Optogenetics has provided an unprecedented opportunity to study somatosensory coding, including nociception. Expressing actuators such as channelrhodopsin-2 (ChR2) in genetically defined subsets of afferents allows those afferents to be selectively activated or inhibited with light applied through the skin (transcutaneously) or directly to the nerve or spinal cord using more invasive methods.^26^^,^^27^ Afferents can be optogenetically activated in combinations not possible with somatosensory stimulation; for instance, mechanical stimuli that activate Aδ high-threshold mechanoreceptors (HTMRs) normally also activate low-threshold mechanoreceptors (LTMRs), so it is only by expressing ChR2 selectively in HTMRs that HTMRs can be activated in isolation.^28^ Causal relationships between afferent co-activation patterns and perception/behavior^29^ can be thoroughly tested in this way. Elucidating those relationships is key to understanding physiological pain and how pathology disrupts normal coding. Optogenetics has been used for basic pain research but, despite its potential, has not yet been adopted for drug testing.^30^ Transcutaneous photostimulation is amenable to high-throughput testing but, like tactile and thermal stimuli, is hard to apply reproducibly in behaving animals.

We sought to improve preclinical pain testing by developing a device able to deliver optogenetic, thermal, and mechanical (tactile) stimuli consistently and measure withdrawal latency automatically with millisecond precision. Using this device, we show that withdrawal latency correlates inversely with the intensity of optogenetic pulses, and that optogenetic ramps reveal differences not seen with pulses. With a clear view of the mouse from below, a neural network was trained to recognize the paw and aim the stimulator, thus fully automating the testing process. The substage video provides a wealth of data about non-reflexive behaviors for consideration alongside withdrawal measurements to more thoroughly assess the pain experience.

## Results

**Figure 1:**
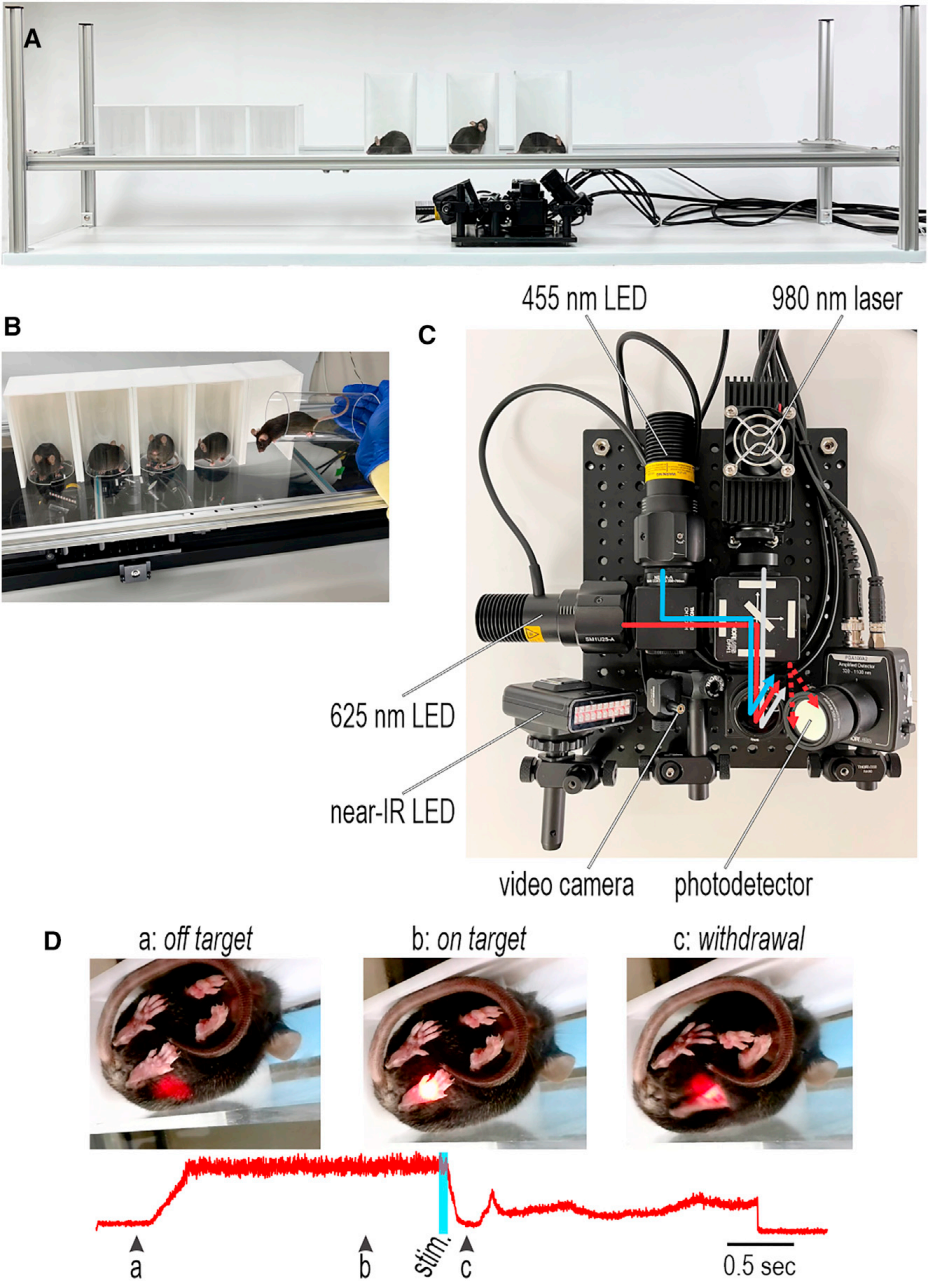
Overview of equipment (A) Mice are kept separately in enclosures on a plexiglass platform (or on a metal grate for mechanical stimulation; see Figure 5B). The stimulator remains at a fixed distance below the platform and is positioned by hand (or by motorized actuators; see Figure 7). (B) Enclosure design. Mice are transferred from their home cage to the platform in a clear plexiglass tube, which is placed vertically on the platform and slid into an opaque cubicle. (C) Top view of photostimulator. Blue and IR light is used for optogenetic stimulation and radiant heating, respectively. Red light is used to help aim and to detect paw withdrawal. All wavelengths are combined and delivered to the same spot, but their intensities are independently controlled by computer. See Figure S1 for details. (D) Sample frames from substage video (Video S1) before the stimulator is properly aimed (a), after aiming (b), and after paw withdrawal (c). Red trace shows the intensity of reflected red light measured by the photodetector. See also Table S1 and Video S1.

Mice are kept individually in enclosures on a clear platform with the stimulator underneath (Figure 1A). A wire grate floor is used when testing mechanical stimuli (see Figure 5B). Different enclosures were tested including a newly developed design in which the mouse is transferred from its home cage in a clear plexiglass tube, which is then turned vertically and slid into an opaque ceilinged cubicle for testing (Figure 1B). Tube/tunnel handling is less stressful than other handling methods.^31^^,^^32^^,^^33^ For high-speed video, which required the mouse to face left to view the stimulated paw in profile, we used a narrow rectangular chamber with clear walls on the front and left side.

Figure 1C shows the stimulator viewed from above. Blue light for optogenetic activation using ChR2, infrared (IR) light for thermal stimulation (radiant heating), and red light for aiming and response measurement are combined into a single beam using dichroic mirrors (Figure S1). The beam is directed vertically and focused to a spot 5 mm in diameter on the platform above. An adjacent camera collects video from below (substage), while a photodetector measures red light reflected off the paw (Figure 1C). A near-IR light-emitting diode (LED) helps improve lighting during high-speed video. The stimulator is translated manually or by motorized actuators (see Figure 7) using substage video to aim. For mechanical stimulation, a computer-controlled indenter is positioned below the wire grate floor (see Figure 5B) but manual or motorized/automated aiming is the same as described above.

As all wavelengths converge on the same spot, red light is turned on prior to initiating photostimulation (with blue or IR light) to verify where photostimuli will hit, thus providing visual feedback to optimize aiming (Figure 1D; Video S1). Rodents are typically assumed not to see red light^34^; though some evidence contradicts this,^35^^,^^36^ we never observed any behavioral response to red light, suggesting that the aiming phase does not provide mice any visual cue about the forthcoming photostimulus. Reflectance of red light off the paw is measured by the adjacent photodetector (red trace). Maximization of the reflectance signal can be used to optimize aiming (compare frames a and b). This reflectance signal is stable while the paw and stimulator are immobile but changes when the paw is withdrawn (frame c), thus enabling measurement of withdrawal latency (see Figure 3). Though too slow to accurately measure fast withdrawals, standard video provides a visual record to rule out gross errors in reflectance-based latency measurements and enables assessment of slower behaviors (see Figure 6).

Video S1. Red light during aiming and paw withdrawal, related to Figure 1

### Reproducible stimulation

**Figure 2:**
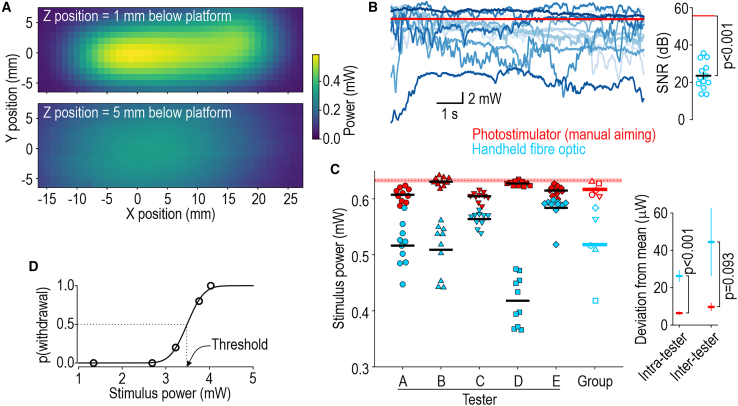
Reproducible photostimulation For (A)–(C), on-target light was measured by stimulating a photodiode facing down on the platform with a paw-shaped cut-out over its surface. (A) Importance of fiber optic positioning. Fiber optic was mounted on linear actuators to control x-y position; the x axis aligns with the long axis of the paw, with 0 position centered on the maximal response. Measurements were repeated for two distances (z) below the platform. (B) Stability of light delivery during 10-s-long photostimuli. Signal-to-noise ratio (SNR = mean^2^/SD^2^) when using the stimulator (55.6 dB; red trace) was significantly higher than for the handheld fiber optic (23.5 ± 2.0 dB, group mean ± SEM; blue traces, 1 for each of 13 testers) (*T*_12_ = 15.8, p < 0.001, one-sample t test). (C) Trial-to-trial variability of light delivery during 100-ms-long pulses. Filled symbols show individual trials with a handheld fiber optic (blue) or stimulator (red) (n = 5 testers, 10 trials/tester/method); black lines represent intra-tester averages. Average trial-to-trial deviation from each tester’s average was significantly larger for handheld fiber optic (26.3 ± 3.0 mW; mean ± SEM) than for photostimulator (6.4 ± 0.7 mW) (*T*_98_ = 6.42, p < 0.001, unpaired t test). Open symbols represent intra-tester averages; colored lines represent group average. Tester-to-tester deviation from group average was larger for handheld fiber optic (44.5 ± 18.2 mW) than for stimulator (9.7 ± 2.0 mW) (*T*_8_ = 1.94, p = 0.093). Red dotted line and shading show average light intensity ± SD across 10 trials without moving the photostimulator. (D) Example input-output curve from one mouse. Five 100-ms-long blue pulses were delivered at each of 5 intensities. Threshold is intensity at 50% probability of withdrawal, as inferred from fitted curve.

Unaccounted for variations in stimulation fundamentally limit the precision with which stimulus-response relationships can be characterized. LEDs and lasers offer stable light sources but the amount of light hitting a target can vary over time or across trials depending on the accuracy and precision of aiming. When applying light by handheld fiber optic (as typically done for transcutaneous stimulation), stability of the tester and differences in aiming technique across testers are important. To gauge the importance of aiming, we measured how the amount of light hitting a target depended on the fiber optic’s positioning in the x-y plane and its distance (z) below the platform. Light was delivered through a paw-shaped cut-out to a photodiode facing downward on the platform (to simulate stimulation of a mouse paw) while controlling fiber optic position with linear actuators. Figure 2A shows that light delivery is sensitive to positioning in all three axes, especially in z (because light rays diverge from the fiber optic tip).

To explore the practical consequences of this, we measured light delivery while 13 testers applied a 10-s-long photostimulus by handheld fiber optic (Figure 2B, blue traces). The signal-to-noise ratio (SNR = mean^2^/SD^2^) of 23.5 ± 2.0 dB (group mean ± SEM) was significantly less than the 55.6 dB obtained with the stimulator (red trace) (*T*_12_ = 15.8, p < 0.001, one-sample t test). The mean stimulus intensity also differed across testers, with an inter-tester coefficient of variation (CV = SD/mean) of 18.8%, which is even larger than the average intra-tester CV of 8.9%. In other words, during a sustained photostimulus, temporal variations in light on target arise from each tester’s instability, but this variability is compounded by differences in aiming technique across testers.

The same issues affect short (pulsed) stimuli but manifest as trial-to-trial variations. To measure variability across trials, five testers used a handheld fiber optic or the photostimulator to deliver ten 100-ms-long pulses to a photodiode (Figure 2C); each pulse was triggered independently. Trial-to-trial deviation of each tester from their individual mean dropped from 26.3 ± 3.0 mW (mean ± SEM) with the handheld fiber optic to 6.4 ± 0.7 mW with the stimulator (*T*_98_ = 6.42, p < 0.001, unpaired t test), which represents a 75.6% reduction in intra-tester variance. Deviation of each tester from the group mean fell from 44.5 ± 18.2 mW with the fiber optic to 9.7 ± 2.0 mW with the photostimulator (*T*_8_ = 1.94, p = 0.093), which represents a 78.2% reduction in inter-tester variance. In other words, using the photostimulator increased reproducibility of stimulation across testers and within each tester.

Even if stimulation is reproducible, behavior is still variable, especially in response to weak stimuli. Threshold is defined as the stimulus intensity at which withdrawal occurs on 50% of trials. Figure 2D shows determination of optogenetic threshold. Reliable aiming combined with precisely controllable LEDs (whose output can be varied in small increments over a broad range) allows one to measure threshold and characterize the broader stimulus-response relationship, assuming responses can be measured precisely.

### Precise response measurement

**Figure 3:**
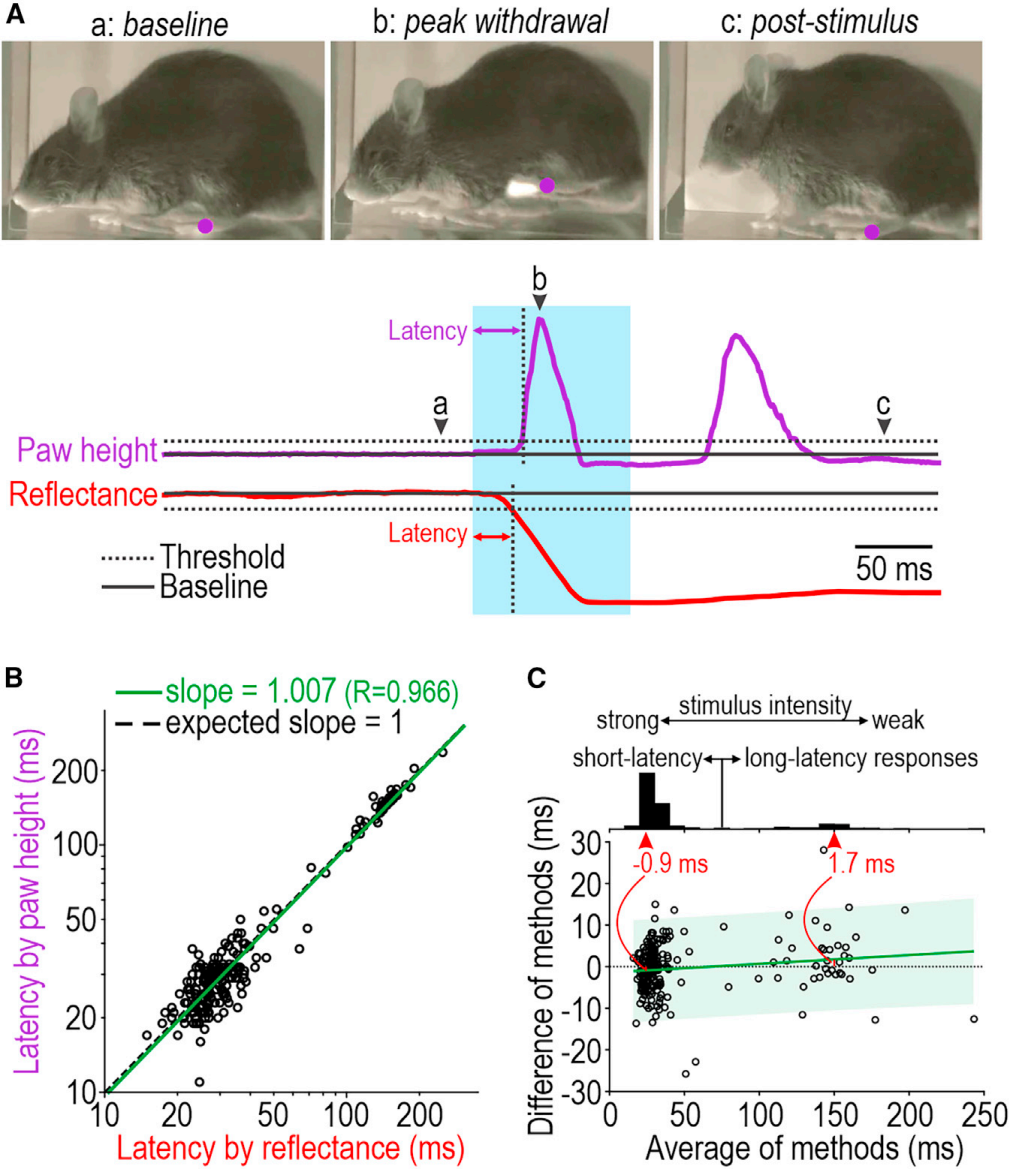
Precise measurement of withdrawal latency (A) Paw withdrawal latency was measured by two methods. Paw height (purple) was extracted from high-speed video (1,000 fps) and is plotted alongside intensity of reflected red light (red) measured by the substage photodetector (1 kHz). Sample frames are shown before (a), during (b), and after (c) stimulation with position of stimulated paw (as tracked by DeepLabCut) summarized by a purple dot (Video S2). Withdrawal latency was determined as delay from stimulus onset until signal crossed threshold (dotted line) defined relative to baseline (solid line) (see STAR Methods). The same threshold value was used for all trials. (B) Comparison of latencies measured from each signal. Data points, each representing a single trial, fell along a line representing equivalence (dashed, slope = 1), yielding a regression line (green) with slope = 1.007. Data are shown on a log scale. Data here are from 6 Advillin-ChR2 mice each tested with a range of stimulus intensities. Starting with 218 trials, 7 trials were excluded (3.2%) based on errors identified by visual inspection of raw data (see Figure S2). (C) Bland-Altman plot using data from (B). For each trial, the difference in latency between methods (i.e., reflectance − paw height) is plotted against the average across methods. The average difference of −0.4 ms does not deviate significantly from 0 (*T*_210_ = −0.8992, p = 0.374, one-sample t test), meaning that there is no fixed bias. Green line and shading show regression line and 95% prediction band. Its slope (0.021) deviates significantly from horizontal (*T*_209_ = 2.31, p = 0.021), suggesting a proportional bias, but the errors are inconsequential: for a short-latency response of 25 ms, latency by reflectance is on average 0.9 ms shorter than latency by paw height (a 3.6% error) whereas for a long-latency response of 150 ms, latency by reflectance is on average 1.7 ms longer (a 1.1% error) (see red highlights). Histogram shows bimodal distribution of latencies and the relation with stimulus intensity (see Figure 4). See also Video S2.

High-speed video is the gold standard for measuring fast behaviors, but acquiring and analyzing those data are complicated and costly. We sought to replace high-speed video by detecting changes in the amount of red light reflected off the paw (see Figure 1D) using a low-cost photodetector. To validate our method, response latency was determined from high-speed video for comparison with latency determined from the reflectance signal on the same trials (Figure 3A). The stimulated paw was identified (purple dot in sample frames) using DeepLabCut^37^ and paw height was measured from each frame (Video S2). Latency was determined independently for each signal based on the time taken for that signal to cross a threshold defined relative to the pre-stimulus baseline (see STAR Methods); after choosing a threshold value in pilot tests, the same value was applied for all subsequent measurements. Each data point in Figure 3B shows the reflectance-based and height-based latency measurement from a single trial plotted relative to one another; data are from 6 mice given 100-ms-long blue pulses with intensities spanning a broad range. The regression line (green, slope = 1.007) follows the equivalence line (dashed, slope = 1). Transforming these data to a Bland-Altman plot (Figure 3C) shows there is no fixed bias and that any proportional bias is inconsequential. Furthermore, the error rate is low (<2%) for each method (Figure S2). Beyond avoiding an expensive high-speed camera and the challenges of filming the mouse in profile to assess paw height, the reflectance signal can be processed in real time to enable closed-loop (automated) termination of photostimuli once paw withdrawal is detected; this was implemented in most experiments using prolonged stimuli, as noted in relevant figure legends. Though not essential for latency measurements, high-speed video can provide additional information.^24^

Video S2. High-speed video of mouse in profile to measure paw height, related to Figure 3

### Characterizing stimulus-response relationships

**Figure 4:**
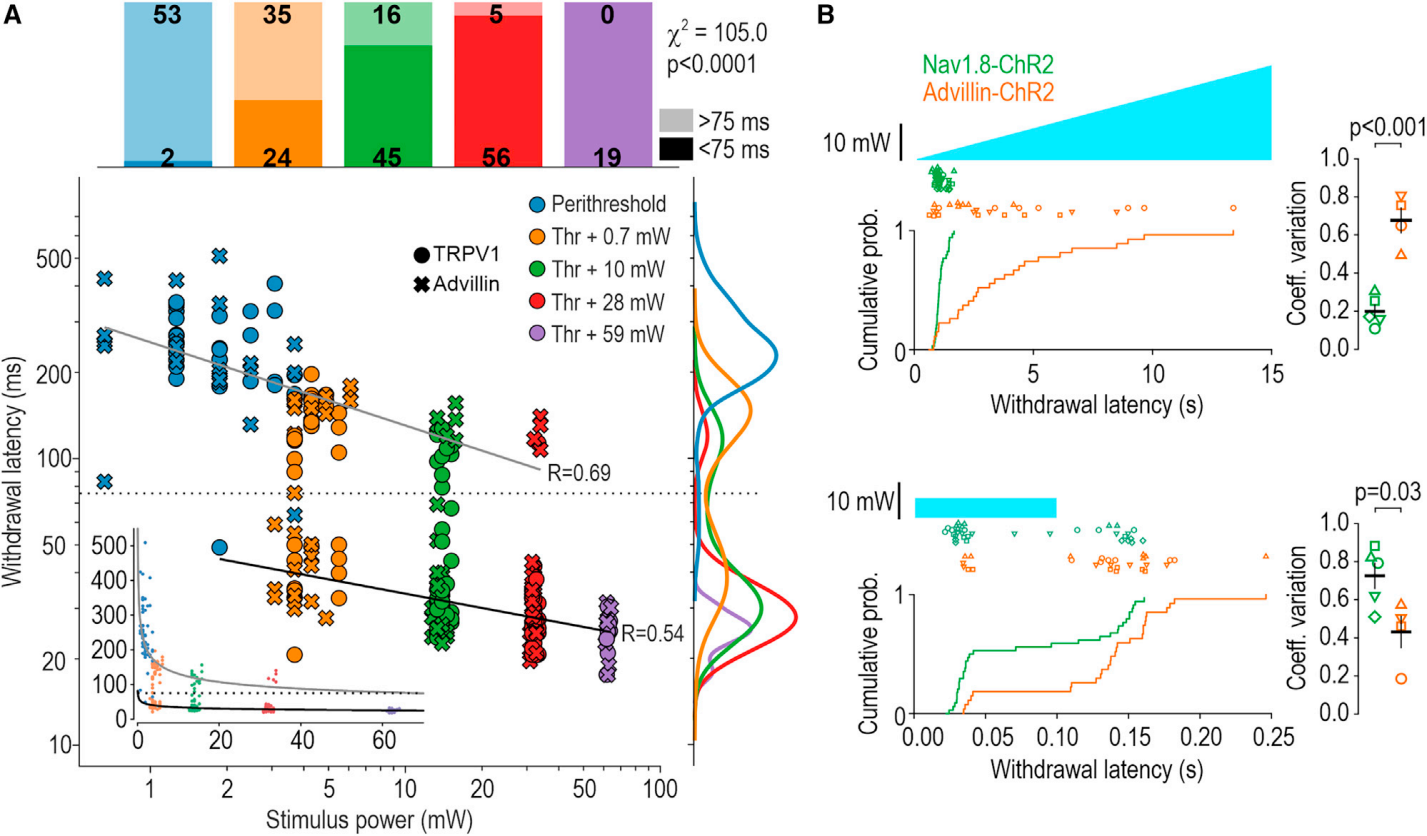
Stimulus-response characterization (A) Impact of photostimulus power. Threshold intensity for 100-ms-long blue photostimuli was determined in 5 Advillin-ChR2 mice (crosses) and 5 TRPV1-ChR2 mice (circles) by two experimenters. Photostimuli were applied at increments above threshold (3 trials/mouse/suprathreshold intensity except for highest intensity, which was tested only once) and withdrawal latency was recorded. Horizontal variations in blue data points reflect intensities used for threshold determination (and are accentuated by the log scale); horizontal variations for other colors reflect inter-animal variability in threshold. Inset shows data on linear scales. Latencies exhibit a bimodal distribution (see histogram on right) with a preponderance of slow (>75 ms) responses at low intensities and fast (<75 ms) responses at high intensities (see stacked bars at top); proportions varied significantly with stimulus intensity (χ^2^ = 105.01, p < 0.0001). Black and gray lines show separate linear regressions for fast and slow responses, respectively, and correspond to exponential curves on linear scales (see inset). Fast, y = 49.8x^−0.17^. Slow, y = 254.8x^−0.29^. (B) Photostimulus ramps. Na_V_1.8-ChR2 mice (n = 5, green) and Advillin-ChR2 mice (n = 4, orange) were tested (7 trials/mouse) with 15-s-long ramps (top; Video S3). Each mouse is represented by a different symbol. Latencies are summarized by their cumulative probability distribution. Ramp-evoked responses were significantly more variable in Advillin-ChR2 mice (*D* = 0.714, p = 3.67 × 10^−8^, two-sample Kolmogorov-Smirnov test). Variability occurs within each mouse; intra-mouse coefficient of variation (=SD/mean) was significantly higher in Advillin-ChR2 mice (*T*_7_ = −6.575, p < 0.001, two-sample t test). Insets report mean ± SEM. Unlike their dissimilar responses to ramps, both genotypes exhibited a similar bimodal latency distribution to pulses (bottom). Distributions differed significantly between genotypes (*D* = 0.403, p = 0.009) but primarily due to the different ratio of short- and long-latency responses. Intra-mouse coefficient of variation was high in both genotypes, but slightly higher in Nav1.8-ChR2 mice (*T*_7_ = 2.71, p = 0.030). See also Video S3.

Minimizing variability in stimulus delivery and response measurement maximizes discrimination of small biological differences; indeed, an input-output relationship is obscured by poorly controlled input or poorly measured output adding noise respectively to the x- and y-positions of constituent data points. To explore how well our device reveals stimulus-dependent variations in withdrawal latency, we titrated the intensity of 100-ms-long pulses of blue light to determine the optogenetic threshold in each of 10 mice. Then, using intensities at defined increments above each mouse’s threshold, we measured withdrawal latency as a function of photostimulus intensity (Figure 4A). Responses evoked by near-threshold intensities (blue) occurred with long latencies (>75 ms), but small increments in intensity (orange and green) evoked responses whose latencies were bimodally distributed and larger increments (red and purple) evoked short-latency (<75 ms) responses. The proportion of slow and fast responses varied significantly with photostimulus intensity (χ^2^ = 105.0, p < 0.0001, excluding purple data points). Browne et al.^38^ reported a similar bimodal distribution of latencies but did not relate this to photostimulus intensity; instead, fast or slow responses occurred randomly in their experiments, perhaps because their ultra-short pulses (3 ms) activated neurons more variably despite their high intensity (47 mW/mm^2^, which is >100 times stronger than thresholds we measured using 100-ms-long pulses). Response latencies within each group decreased with increasing photostimulus intensity.

To date, all reports of withdrawal from transcutaneous optogenetic stimulation used a pulse of blue light or pulse trains,^28^^,^^38^^,^^39^^,^^40^^,^^41^^,^^42^^,^^43^^,^^44^^,^^45^^,^^46^^,^^47^^,^^48^^,^^49^ with one exception, which used sustained light to activate keratinocytes.^50^ Yet different photostimulus waveforms may reveal different information about the neural control of behavior (see discussion), so testing with different waveforms will provide greater information than testing with any one waveform. Therefore, capitalizing on the stability of our stimulator (see Figure 2B), we tested slowly ramped photostimuli (and pulses) in two transgenic mouse lines: Advillin-ChR2 mice express ChR2 in all somatosensory afferents,^51^ whereas Na_V_1.8-ChR2 mice express ChR2 selectively in nociceptors.^52^^,^^53^ Whereas Na_V_1.8-ChR2 mice responded consistently with a latency of 1.1 ± 0.2 s (mean ± SD), Advillin-ChR2 mice responded with much longer latencies on some trials (Figure 4B, top). The difference was due mostly to intra-mouse variability, with individual Advillin-ChR2 mice responding with a broad range of latencies rather than some mice being consistently slow and others being consistently fast. By comparison, both genotypes exhibited a similar bimodal latency distribution when tested with pulses (Figure 4B, bottom). Latencies are nearly three orders of magnitude slower for ramp-evoked responses than for pulse-evoked responses, meaning “slow” pulse-evoked responses are still much faster than “fast” ramp-evoked responses. Our goal here was not to compare pulse and ramp stimuli but, rather, to show that one stimulus waveform might reveal differences (e.g., between genotypes) that are not revealed by other waveforms, attesting to the value of testing with different stimulus kinetics in addition to different stimulus intensities (see Figure 4A) and modalities (see below).

### Additional stimulus modalities

**Figure 5:**
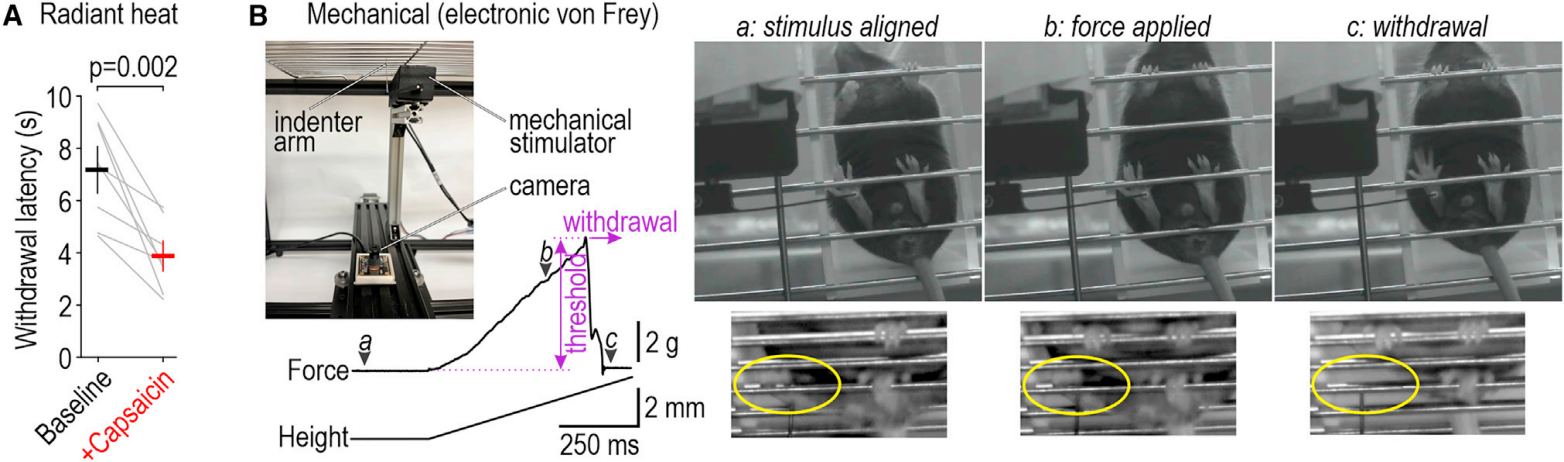
Additional stimulus modalities (A) Thermal stimulation. Radiant heat was applied via infrared laser (Video S4). Latency dropped from 7.18 ± 0.74 s (mean ± SEM) at baseline to 3.88 ± 0.49 s after injecting 0.5% capsaicin into the left hind paw (*T*_7_ = 4.64, p = 0.002, paired t test, n = 8 mice, 3 trials/mouse for baseline, 3–5 trials/mouse for +capsaicin). Thermal stimulation was automatically terminated after detection of withdrawal or after 20 s cutoff. (B) Mechanical stimulation. Top left image shows configuration of equipment. Tip of indenter arm is aimed using substage video. Height of indenter arm is ramped up by computer control while monitoring (at 1 kHz) the force exerted on the paw. Withdrawal causes an abrupt drop in force. Threshold is taken as the peak force preceding withdrawal. Sample frames extracted from the standard-rate video used for aiming (top) and the high-speed (1,000 fps) video used for validation (bottom) illustrate aiming (a), stimulation (b), and withdrawal (c) phases (Video S5). Yellow highlights the target paw, which has moved out of view in (c). See also Videos S4 and S5.

Despite focusing hitherto on optogenetic stimuli, our device can deliver more conventional stimuli and automatically measure withdrawal. Radiant heat is applied with an IR laser (see Figure 1C). Laser intensity was adjusted in pilot experiments to evoke withdrawal after ∼8 s, as in a standard Hargreaves test. Heating was automatically terminated upon detection of paw withdrawal or after a 20 s cutoff. Withdrawal latency was significantly reduced after injecting 0.5% capsaicin into the hind paw (Figure 5A; Video S4). Mechanical stimulation is applied with a computer-controlled, force-feedback indenter (Figure 5B, inset). With the mouse positioned on metal grate floor (instead of plexiglass), the indenter tip is aimed via substage video before being raised at a fixed rate (Figure 5B; Video S5). Withdrawal is evident from the rapid drop in force as sensed by the indenter (at 1 kHz) and verified by high-speed video (at 1,000 fps), thus precluding the need for reflectance-based latency measurements. Mechanical threshold is taken as the peak force immediately prior to withdrawal.

Video S3. Responses to photostimulus pulse and ramp, related to Figure 4

**Figure 6:**
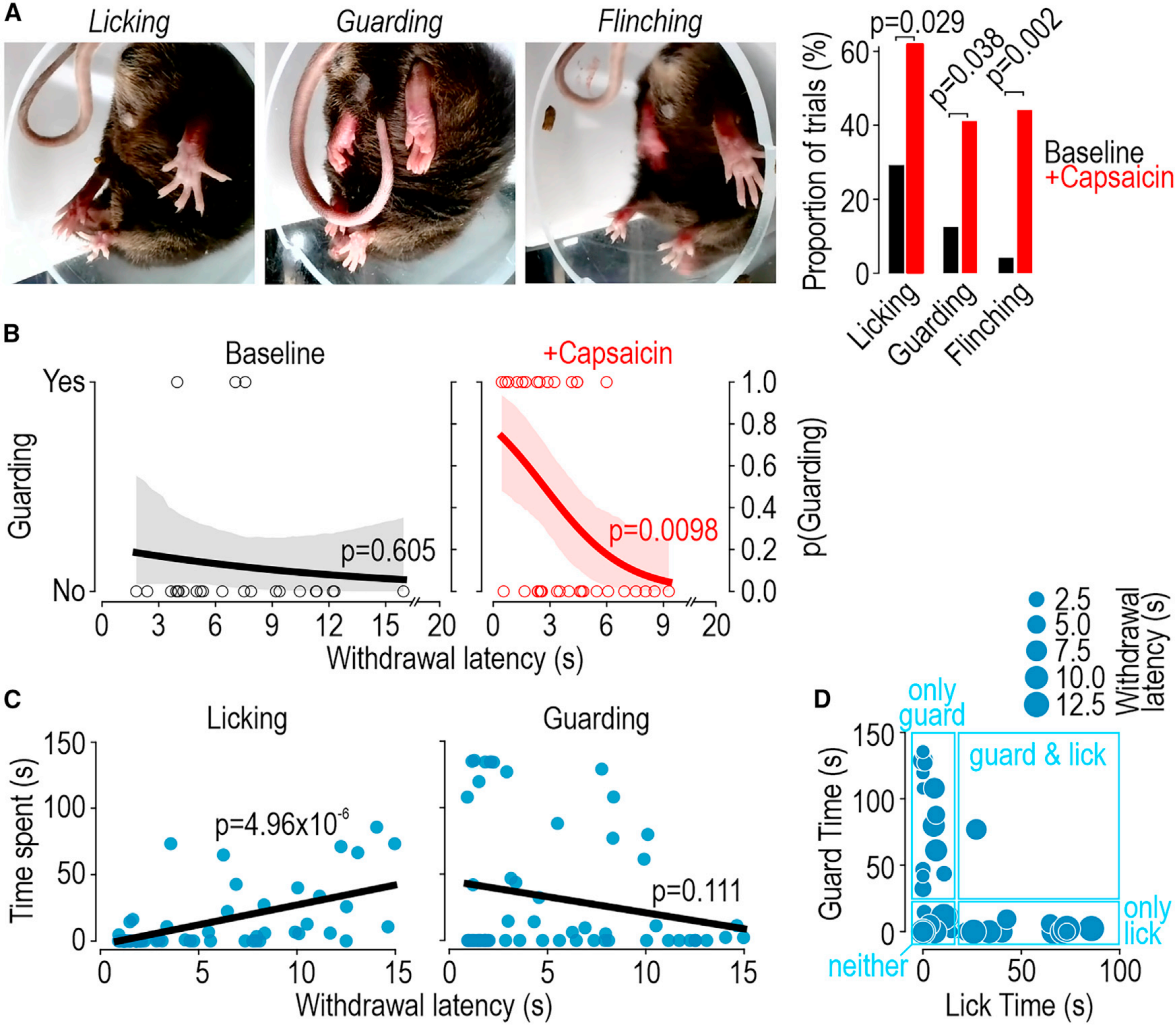
Additional behavior measurements (A) Non-reflexive behaviors after radiant heat. Sample frames from Video S4 illustrate licking, guarding, and flinching (same experiments reported in Figure 5A). These behaviors were significantly more common after capsaicin (red; n = 34 trials) than at baseline (black; n = 24 trials). p values on graph report χ^2^ tests. These behaviors were rare in the pre-stimulus period. (B) Correlation between guarding and withdrawal latency. Extending analysis in (A), occurrence of guarding on a given trial (yes/no) was plotted against withdrawal latency. According to logistic regression, guarding was significantly more likely after shorter latency responses in the +capsaicin condition (p = 0.0098) but not at baseline (p = 0.605). Shading indicates bootstrapped 95% confidence interval. See Figure S3 for analysis of licking and flinching. (C) Non-reflexive behaviors after optogenetic ramps. In 5 TRPV1-ChR2 mice given 15-s-long optogenetic ramps (9–12 trials/mouse), withdrawal latency was plotted against the time spent licking or guarding during the subsequent post-withdrawal period (2 min duration + time remaining in ramp after withdrawal). p values on graphs show strength of correlation, which trended in opposite directions. Optogenetic stimulation was automatically terminated after detection of withdrawal. (D) Guarding vs. licking. Plotting guard time against lick time on a trial-by-trial basis reveals that mice typically exhibit one or the other behavior on a given trial, not both. See also Video S4.

Video S4. Responses to radiant heat before and after intraplantar capsaicin, related to Figures 5 and 6

Video S5. Automated mechanical stimulation and response measurement, related to Figure 5

### Additional response measures

For all stimulus modalities, the substage video enables slower, non-reflexive behaviors to be analyzed. For example, video of withdrawals reported in Figure 5A revealed that thermal stimulation triggered significantly more licking, guarding, and flinching after capsaicin (Figure 6A). By plotting the occurrence or absence of these non-reflexive behaviors against latency of the preceding withdrawal, logistic regression revealed that guarding was not correlated with withdrawal latency under baseline conditions but, after capsaicin, was significantly more likely following short-latency withdrawals (Figure 6B) (logistic regression, p = 0.605 on the basis of 22 baseline trials vs. p = 0.00985 on the basis of 32 +capsaicin trials). Equivalent analysis for licking and flinching is reported in Figure S3. One may cautiously interpret this to mean capsaicin causes heat to be perceived as more painful, triggering faster withdrawal, whereas short-latency responses occasionally occur under baseline conditions but not because certain trials are more painful than other trials, providing clues as to where variability arises.^54^

We similarly analyzed responses to optogenetic ramps because the high intra-mouse variability in withdrawal latency affords an ideal opportunity to test if short- or long-latency withdrawals in the same mouse are more or less painful. Plotting the amount of time spent licking or guarding (during a ∼2 min post-stimulus period) against withdrawal latency (Figure 6C) revealed that long-latency withdrawals were associated with significantly more licking (*T*_55_ = 5.06, p = 4.96 × 10^−6^, one-sample t test on slope) but not more guarding (*T*_55_ = −1.62, p = 0.111). This suggests that failure to withdraw promptly, for reasons that remain unclear, results in the stimulus causing more pain, as inferred from licking. Interestingly, plotting time spent licking against time spent guarding on a trial-by-trial basis shows that mice tend to exhibit one or the other behavior on a given trial (Figure 6D). Interpretations warrant caution but automated analysis can expedite and help standardize future investigation along these lines.

### Fully automated testing

**Figure 7:**
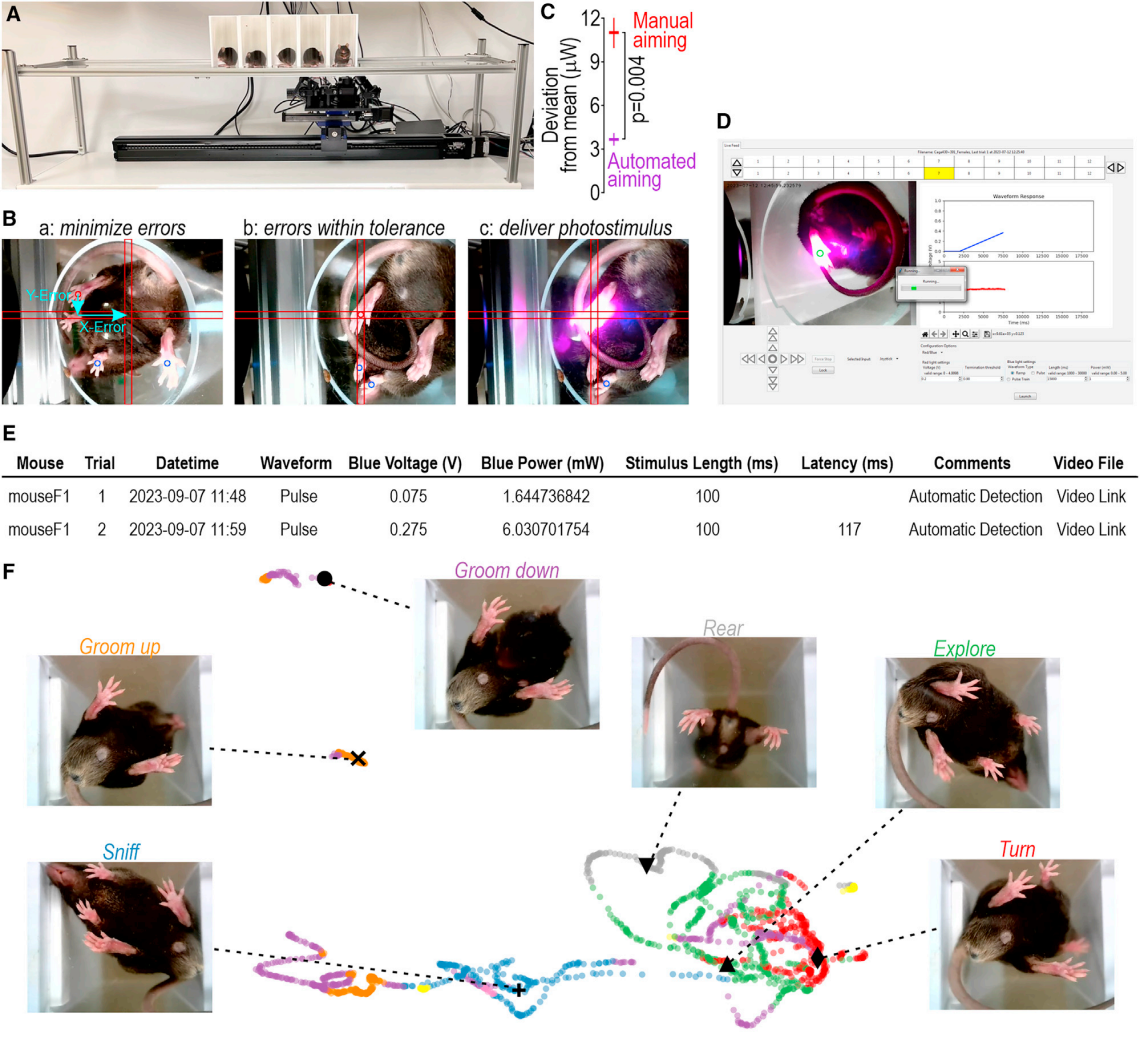
Fully automated testing (A) Motorized photostimulator. Unlike the manually aimed version (see Figure 1), this version is mounted on linear actuators that allow its computer-controlled translation in the x and y axes. Aiming is controlled by joystick or automatically with machine learning. (B) Automated aiming. A neural network identifies the paws, snout, and tail base from substage video. Deviation of the target paw from the crosshairs is measured (a) and then minimized by translating the stimulator (b). In other words, x- and y-errors are reduced until the center of the target paw (red dot) is within 3 pixels of the screen center, which is aligned with the stimulation zone. Once the paw has remained stable inside the crosshairs for a minimum (user-defined) period, photostimulation is initiated (c), and withdrawal latency is measured and recorded. The stimulator then moves to the adjacent mouse. See Video S6 for an example of fully automated testing with radiant heat. Automated aiming is also compatible with the wire grate floor used for mechanical stimulation (Video S7). (C) Automated aiming (10 trials), quantified as in Figure 2C, was significantly less variable than manual aiming (5 testers, 10 trials each) of the photostimulator (*T*_58_ = 3.03, p = 0.004, unpaired t test). Stability of prolonged photostimulation (cf. Figure 2B) is equivalent regardless of aiming method. Graph reports mean ± SEM. (D) Graphical user interface. Figure S4 shows enlarged view with description. (E) Sample of spreadsheet to all which data and metadata from each trial is automatically saved with linked graphs and videos. See Data S1 for a sample spreadsheet with data and linked videos for fully automated testing of optogenetic pulses and ramps. (F) Automated, non-supervised classification of ongoing behavior. Colored dots show latent space embedding of behavioral state determined at regular intervals from Video S8. Screenshots corresponding to labeled points illustrate specific behaviors. See also Data S1 and Videos S6, S7, and S8.

Next, we mounted the stimulator on linear actuators (Figure 7A) so that aiming could be controlled remotely by joystick (i.e., without the tester operating in close proximity to the mice) or automatically using machine learning. For the latter, a neural network was trained with substage video using DeepLabCut to recognize the paws and other points on the mouse. The computer is then fed substage video and DeepLabCut-Live^55^ uses the trained network to position the photostimulator by minimizing x- and y-error signals (to within 3 pixels) so that the target paw is positioned in the crosshairs for stimulation (Figure 7B; Video S6). Stimulation initiates automatically once the paw has remained stationary for a minimum period and terminates automatically upon detection of paw withdrawal or after a pre-set cutoff. Automated aiming delivered stimuli even more reproducibly than manual aiming with the same device (Figure 7C). This testing was conducted using a neural network trained to recognize the paw-shaped cut-out over the photodiode; neural networks trained to recognize real paws may differ in performance, though post hoc analysis of video records suggests excellent performance identifying the center of the paw and targeting that point for stimulation. For mechanical stimulation, the metal grate floor partially obscures the mouse but automated aiming is still possible with an appropriately trained neural network (Video S7).

Video S6. Fully automated testing with radiant heat, related to Figure 7

Video S7. Automated aiming of mechanical stimulator, related to Figure 7

After completing a trial, the device automatically proceeds to the neighboring mouse. By interleaving trials, other mice are tested during the inter-stimulus interval required for each mouse, thus expediting the overall testing process. The order of testing can easily be randomized, which is difficult for an experimenter to keep track of. Meta-data (mouse identification, date, time, stimulus parameters), raw data (video, reflectance signal) and measurements (latency, threshold) are automatically saved (Figure 7E; Data S1). Non-reflexive behaviors such as those analyzed in Figure 6 can also be detected and quantified automatically using unsupervised methods (Figure 7F; Video S8). Although not yet implement, automated real-time classification of posture can be integrated into withdrawal testing so that stimuli are applied contingent on certain postures; for example, guarding and rearing can influence withdrawal latencies^38^^,^^56^ and stimulation could, therefore, be delayed until the mouse assumes a preferred posture. Even without closed-loop control, video records enable post hoc correlation of withdrawal latency with the pre-stimulus posture. Standardized high-throughput testing without the potential errors, systematic differences,^19^ and animal stress^57^ associated with human testers is thus realized.

Video S8. Ongoing behavior and its automated classification, related to Figure 7

## Discussion

We developed a device able to reproducibly deliver photostimuli of different wavelengths, intensities, and kinetics (waveforms). A photometer detects paw withdrawal and measures withdrawal latency with millisecond precision based on changes in the reflectance of red light. The accuracy of this approach was validated by comparison with high-speed video. Closed-loop control of stimulation is made possible by real-time detection of paw movement. We also demonstrate computer-controlled mechanical stimulation and automated detection of touch-evoked withdrawal. Building on computer-controlled stimulation and response measurement, we automated video-based aiming by using neural networks to track the paw plus motorized actuators to move the stimulator. Whereas aiming by joystick prevents the tester from working in close proximity to the mice, which stresses them,^57^ automation removes the human element altogether, with significant benefits for standardization, objectivity, and throughput. Video records allow non-reflexive behaviors to be quantified and correlated with withdrawal measurements. Automation also facilitates standardized recording of data and metadata, which is crucial for creating large datasets amenable to mining.

Capitalizing on identification of gene expression patterns to distinguish subtypes of somatosensory afferents,^58^ optogenetics affords an unprecedented opportunity to study somatosensory coding by activating or inhibiting specific afferent subtypes. Testing the behavioral response to synthetic activation patterns allows one to explore causal relationships, complementing efforts to characterize co-activation patterns evoked by natural stimuli.^59^ This does not require that optogenetic stimuli mimic natural, somatosensory stimuli. Optogenetic stimuli are patently unnatural—and the evoked sensations probably feel unnatural, like paresthesias evoked by electrical stimulation—but their ability to evoke behavior allows one to start inferring how they are perceived, and how those sensations relate to neural activation patterns. Doing this requires tight control of the stimulus and precise measurement of the response.

Technical advances have been made in delivering photostimuli to the CNS or peripheral nerves for optogenetic manipulations.^60^ This technology is invaluable, but stimulating the nerve does not reproduce somatotopically organized stimulation. Photostimulating receptive fields in the skin is preferable in that regard, and is also less invasive. However, transcutaneous stimuli are difficult to apply reproducibly to behaving animals. Sharif et al.^48^ solved this by mounting the fiber optic to the head to stimulate the cheek, but a comparable solution is infeasible for stimulating paws. These technical challenges explain why past studies focused on whether mice responded to optogenetic stimulation, without carefully varying stimulus parameters or measuring subtler aspects of the response. Past studies have varied the number, rate, or intensity of pulses, but in the suprathreshold regime, with consequences for the amount of licking, jumping, or vocalization. To our knowledge, only one study^61^ titrated the intensity of transcutaneous photostimuli to determine threshold (see Figure 2D), and another^43^ titrated pulse duration and spot size. Moreover, scoring responses by eye, though still the norm for many tests, must be replaced with objective metrics. Schorscher-Petcu et al.^43^ recently described a device that uses galvanometric mirrors to direct photostimuli and high-speed substage video to measure withdrawal. Their device is very elegant, but reliance on high-speed video to detect withdrawals likely precludes closed-loop control, and nor is their device fully automated or high-throughput. Our device delivers reproducible photostimuli and automatically measures withdrawals using the red-reflectance signal complemented by regular-speed substage video, which is also used for automated aiming.

Non-painful stimuli may trigger withdrawal, which is to say that the threshold stimulus (or the probability of withdrawal) may not reflect painfulness.^24^ In that respect, testing with stronger stimuli is informative. The study by Browne et al.^38^ stands out for its use of high-speed video to thoroughly quantify responses to optogenetic stimulation. Like us (see Figure 4A), they observed a bimodal distribution of withdrawal latencies; however, they observed this despite using high-intensity pulses, most likely because their pulses were extremely brief (3 ms) and might, therefore, have activated afferents probabilistically. By varying the intensity of longer (100 ms) pulses, we observed that stronger stimuli evoke faster withdrawals, evident as a continuous shift in latency as well as a switching from long- to short-latency responses. A putative explanation for the bimodal latency distribution—consistent with Browne et al.^38^ and with the double-alarm system proposed by Plaghki et al.^62^ based on different rates of heating—is that slow and fast responses are mediated by C- and A-fibers, respectively. Building from that, our data suggest that C-fibers are recruited first (i.e., by weaker photostimuli) and that slow responses speed up as more C-fibers get recruited, but a discontinuous “switch” to fast responses occurs once A-fibers get recruited, and fast responses speed up as more A-fibers are recruited. Further investigation is required but resolving the stimulus-response relationship sufficiently to even pose such questions is notable.

Browne et al.^38^ also noted that the withdrawal response was not limited to the stimulated limb and, instead, was more widespread. Although not quantified here, a widespread response was evident in substage video and sometimes included vocalizing, facial grimacing, jumping, and orienting to the stimulus followed by licking, guarding, or flinching of the stimulated paw (see Figure 6). A complete analysis of each trial ought to consider not only the reflexive component (i.e., did withdrawal occur and how quickly), but also whether signs of discomfort were exhibited afterward and for how long. Those signs are obvious when applying strong stimuli but become harder to discern with near-threshold stimuli, which makes objective quantification all the more important. Regular-speed video is sufficient to capture all but the fast reflex (which can be measured by other means; see Figure 3) and the bottom-up view is well suited for machine-learning-based quantification of ongoing behaviors.^63^^,^^64^ We recommend that video be recorded for all trials if only to allow analysis of those data in the future. There has been an explosion of artificial intelligence (AI)-based methods for extracting key points on animals^37^^,^^65^^,^^66^ and algorithms for extracting higher-level behaviors from key point^63^^,^^64^^,^^67^ or raw pixel^68^ data. Application of such tools is yielding impressive results.^69^ Other hardware has been recently developed to facilitate such analysis but does not include stimulation capabilities.^70^ By capturing video before and after stimuli, our device enables users to quantify behaviors in addition to measuring reflexive withdrawal using traditional metrics.

Withdrawal responses are known to be sensitive to posture and ongoing behavior at the time of stimulation.^38^^,^^56^^,^^71^ Such differences may confound latency measurements but may also provide important information; either way, they should be accounted for. Waiting for each mouse to adopt a specific posture is onerous for a human tester but can be done by computer if automated stimulation is made contingent on the mouse being in a certain state. Before that, to better understand the relationship between the mouse’s pre-stimulus state and its subsequent stimulus-evoked withdrawal (and post-stimulus state), its state at stimulus onset could be classified from video (like in Figure 7F) and correlated with the evoked response on that trial (as for post-stimulus behaviors in Figures 6B and 6C). Other comparisons would also be informative, like correlating the pre- and post-stimulus states and treatment status. In short, more data can be acquired and more thoroughly analyzed than is typically done in current protocols; others have also advocated for this.^23^^,^^24^^,^^25^ More comprehensive analysis need not entail expensive equipment or reduced throughput.

Nearly all past studies involving transcutaneous optogenetic stimulation used single pulses or pulse trains.^28^^,^^38^^,^^39^^,^^40^^,^^41^^,^^42^^,^^43^^,^^44^^,^^45^^,^^46^^,^^47^^,^^48^^,^^49^ In the one exception, Baumbauer et al.^50^ activated ChR2-expressing keratinocytes with sustained light. Single pulses and pulse trains are just some of the many possible waveforms, especially as LEDs can be so easily controlled. Notably, just like pulsed electrical stimuli lost favor in pain testing because of the unnaturally synchronized neural activation they evoke,^1^ pulsed optogenetic stimuli warrant similar scrutiny and should not be the only waveform tested. Indeed, different rates of radiant heating differentially engage C- and A-fibers,^62^ thus enabling the role of different afferents to be studied. By testing photostimulus ramps, we uncovered genotypic differences that were not evident with pulses (see Figure 4B). The basis for the genotypic difference requires further investigation but we hypothesize that co-activation of non-nociceptive afferents in Advillin- and TRPV1-ChR2 mice (but not in Nav1.8-ChR2 mice) engages a gate control mechanism that tempers effects of nociceptive input, consistent with Arcourt et al.,^28^ who showed that activating Aδ-HTMRs in isolation evoked more guarding, jumping, and vocalization than co-activating Aδ-HTMRs and LTMRs. By testing different photostimulus waveforms, one can start to delineate the underlying interactions. Photostimulus kinetics influence how optogenetic actuators such as ChR2 respond (e.g., whether they desensitize, thus producing less current for a given photostimulus intensity), but one must also consider how neurons respond to those photocurrents. Specifically, pulsed stimuli tend to evoke precisely timed spikes,^38^ leading to spikes that are synchronized across co-activated neurons,^72^ which may or may not accurately reflect the spiking patterns evoked by somatosensory stimuli. Artificial stimuli need not mimic natural stimuli to be informative; indeed, deliberately evoking spiking patterns not possible with natural stimuli offers new opportunities to probe somatosensory coding, including the role of synchrony. In that respect, optogenetic testing should include but not be limited to pulsed photostimuli. Interestingly, some studies^44^^,^^45^ have tested if ChR2-expressing mice avoid blue-lit floors, which they do. In these cases, the floor light was continuous, unlike the pulses typically applied by fiber optic; it is therefore notable that mice avoided the blue floor but did not respond to it with reflexive withdrawal, paw licking, or other outward signs of pain, as they did to pulses. In the one case where the floor light was pulsed,^47^ reflexive withdrawal was observed. These results highlight the underappreciated importance of stimulus kinetics.

To summarize, we describe a device capable of reproducible, automated, multimodal algometry, or RAMalgo. Aiming, stimulation, and measurement are fully automated, which improves standardization and increases throughput, among other benefits. A video record of the animal before, during, and after stimulation allows one to extend analysis beyond traditional response metrics (i.e., threshold and latency) to consider if evoked and ongoing pain behaviors are correlated.

### Limitations of the study

Though we have demonstrated computer-controlled mechanical stimulation, we did not compare this against von Frey filaments, for instance, by comparing intra- and inter-tester variability in stimulus delivery like we did for photostimulation. There is still room to improve automated mechanical stimulation and to add other stimulus modalities. Many other conceivable scenarios have not yet been tested; for instance, *in vivo* electrophysiology and calcium imaging could be precisely synchronized with stimulation and behavioral response measurements using this technology. Unlike home-cage monitoring, mice must be transferred to an unfamiliar environment for testing. Best practices must be applied when handling mice, acclimating them, testing at the same time of day, etc., to minimize stress and the variability it introduces.

## STAR★Methods

### Key resources table

**Table:** 

REAGENT or RESOURCE	SOURCE	IDENTIFIER
**Experimental models: Organisms/strains**		

Mouse: Ai32, Cre-dependent ChR2	JAX	RRID:IMSR_JAX:024109
MouseTRPV1 Cre	JAX	RRID:IMSR_JAX:017769
Mouse: Advillin Cre	Zhou et al.^51^	N/A
Mouse: Nav1.8 Cre	Agarwal et al. ^53^	N/A

**Deposited data**		

Data for model training	This paper	Mendeley Data: https://doi.org/10.17632/gn2wbkh7j3.1

**Software and algorithms**		

Computer code	This paper	GitHub: https://github.com/stofe95/ramalgo; Zenodo: https://doi.org/10.5281/zenodo.10022925
Spike2	Cambridge Electronic Design	RRID:SCR_000903
Python 3.8	The Python Software Foundation	https://www.python.org/; RRID:SCR_008394
Matplotlib	The Matplotlib community	https://matplotlib.org/; RRID:SCR_008624
Seaborn	The Seaborn community	https://seaborn.pydata.org/; RRID:SCR_018132
Pandas	The Pandas community	https://pandas.pydata.org; RRID:SCR_018214
Numpy	The Numpy community	https://numpy.org/; RRID:SCR_008633
Scipy	The Scipy community	https://scipy.org/; RRID:SCR_008058
OpenCV	The OpenCV community	https://opencv.org/; RRID:SCR_015526
VAME	Luxem et al. ^64^	https://github.com/LINCellularNeuroscience/VAME; RRID:SCR_022477
DeepLabCut	DeepLabCut developers Mathis et al.^37^	RRID:SCR_021391
DeepLabCut-live	DeepLabCut developers Kane et al.^55^	https://github.com/DeepLabCut/DeepLabCut-live
PyTrinamic	Trynamic Motion Control	https://github.com/trinamic/PyTrinamic

### Resource availability

#### Lead contact

Further inquiries or requests can be directed to Steve Prescott (steve.prescott@sickkids.ca).

#### Materials availability

This study did not generate new unique reagents. A full parts list is provided in Table S1 which, together with details in Figure S1, allows users to construct their own stimulator. The device will also be made commercially available in the near future.

#### Data and code availability

• All data reported in the paper are available upon request. Data used to train the mouse keypoint detection model using DeepLabCut is available at Mendeley Data (https://doi.org/10.17632/gn2wbkh7j3.1). This includes the full output directory from DeepLabCut, including 500 labeled frames used to train a Resnet-50 based model. Model performance depends on various factors (camera angle, lighting, background, etc.) and so performance will differ if those factors differ. Users should add their own training data representative of their testing conditions.
• Original code for running the device will be made available on GitHub upon publication (https://github.com/stofe95/ramalgo). An archival DOI is reported in the key resources table.
• Any additional information required to reproduce the results reported in this study is available from the lead contact upon request.

### Experimental model and subject details

All procedures were approved by the Animal Care Committee at The Hospital for Sick Children (protocol #53451) and were conducted in accordance with guidelines from the Canadian Council on Animal Care. To express ChR2 selectively in different types of primary somatosensory afferents, we used Ai32(RCL-ChR2(H134R)/EYFP) mice (JAX:024109), which express the H134R variant of ChR2 in cells expressing Cre recombinase. These were crossed with advillin^Cre^ mice (kindly provided by Fan Wang) to express ChR2 in all sensory afferents, TRPV1^Cre^ mice (JAX:017769) to express ChR2 in TRPV1-lineage neurons, or Na_V_1.8^Cre^ mice (kindly provided by Rohini Kuner) to express ChR2 in nociceptors. 8–16 week old male (n = 28) or female (n = 11) mice were acclimated to their testing chambers for 1 h on the day before the first day of testing, and each day for 1 h prior to the start of testing. Sex differences were not observed and data were therefore pooled.

### Method details

#### Photostimulator

The stimulator is summarized in Figure S1; A complete list of components (as numbered in Figure S1, with part # and supplier information) is included as Table S1. Briefly, collimated light from a red (625 nm) LED and blue (455 nm) LED is combined using a 550 nm cut-on dichroic mirror. Blue light is attenuated with a neutral density filter. This beam is combined with IR light from a 980 nm solid laser using a 900 nm cut-on dichroic mirror. The IR beam is expanded to fill the back of the focusing lens. The common light path is reflected upward with a mirror and focused to a spot 5 mm in diameter on the platform above. The surface area of the spot is ∼20 mm^2^; photostimulus power values should be divided by this number to convert to light density (irradiance). Red light reflected off the mouse paw is collected by a photodetector through a 630 nm notch filter. All light sources are controlled by computer via appropriate drivers and a 1401 DAQ (Cambridge Electronic Design) using Spike2 (Cambridge Electronic Design) or custom software written in Python using Numpy,^73^ Scipy,^74^ Pandas,^75^ Matplotlib,^76^ and OpenCV.^77^ The photodetector samples at 1 kHz with the same DAQ, thus synchronizing stimulation and withdrawal measurement. A camera provides video of the mouse from below (substage). Video is used for aiming with the help of visual feedback using the red light, which is turned on prior to photostimulation with blue or IR light. A near-IR light source is useful to improve lighting during high-speed video. In the manual version of the device, the device is slid by hand; leveling screws at the four corners of the breadboard have a plastic cap for smooth sliding. In the motorized version, the breadboard it attached to linear actuators (TBI Motion) via 3-D printed connectors. Motors are controlled via custom software. The user aims by keyboard or joystick, or fully automated aiming is left to a neural network trained to recognize the mouse paws.

#### Mechanostimulator

Computer-controlled mechanical stimulation was implemented using a 300C-I dual-mode indenter (Aurora Scientific). This stimulator can control and measure both force and length (height). Our software controls height in the same way LED/laser intensity is controlled for photostimulation. The exerted force is simultaneously measured at 1 kHz and recorded to computer. Because withdrawal is evident from changes in measured force, additional signals (e.g., reflectance, video) are not required for latency measurements.

#### Platform and enclosures

The platform and animal enclosures were custom made. Except when testing mechanical stimuli, the platform is 3 mm-thick clear Plexiglass mounted on 20 × 20 mm aluminum rails, adjusted to the desired height above the stimulator. For mechanical stimulation, plexiglass was replaced with a metal grate, specifically a stainless steel cooling rack. Various enclosure designs were tested. In the final design, clear Plexiglass tubes (outer diameter = 65 mm, thickness = 2 mm) cut in 12.5 cm lengths were used in conjunction with opaque white 3-D printed cubicle. The same tube used to transfer a mouse from its home cage is placed on the platform vertically and slid into a cubicle for testing (see Figure 1B). A notch cut into the base of each tube allows the experimenter to deliver a food reward, to poke the mouse (to wake or orient it), or to clean feces or urine from the platform if required. Each cubicle is 3-D printed and contains internal magnets that allow cubicles to be easily combined. Keeping the mice at fixed distances from each other is important for automated testing, where the stimulator is automatically translated a fixed distance when testing consecutive mice. To view the mouse in profile during high-speed video, we used a narrow rectangular chamber with clear walls on the front and left side (with a notch under the latter) and opaque walls at the rear and right side. In some cases, a mirror was placed at a 45° angle near the left wall to simultaneously capture a front view of the mouse.

#### Comparison with handheld fiber optic

Volunteer testers were instructed to use a fiber optic (multimode fiber optic patch cable, 1000 μm diameter core, NA = 0.48, SMA endings attached to 455 nm fiber-couple LED, Thorlabs) to apply a photostimulus to an s170C photodiode attached to a PM100D optical power meter (Thorlabs). The same photostimulus power was used for all trials, by all testers. The photodiode was covered with a paw-shaped cutout and placed face down on the plexiglass platform to simulate aiming at a real paw standing on the platform. The PM100D output was connected to a Power1401 data acquisition interface (Cambridge Electronic Design), sampling at 1 kHz. The Power1401 was also used to deliver command voltages to the LEDD1B LED driver (Thorlabs).

#### Automated withdrawal detection and latency measurement

Paw withdrawal is detected and its latency measured from the red reflectance signal using custom code written in Python. Red light is initiated prior to photostimulation with blue or IR light. Baseline reflectance is measured over the 0.5 s epoch preceding photostimulus onset. A running average across a 27 ms-wide window was used to remove noise. Withdrawal latency was defined as time elapsed from photostimulus onset until the reflectance signal dropped below a threshold defined as 2 mV below baseline; the signal needed to remain below threshold for >20 ms to qualify as a response, but latency was calculated based on the start of that period. The 2 mV threshold value was chosen based on pilot experiments and then applied unchanged in all subsequent testing. Latencies thus extracted from the reflectance signal were compared to latency values extracted from high-speed video of the same withdrawal. In the latter case, paw height was extracted from video (see below) using DeepLabCut; latency was taken as the time taken for paw height to rise 6 pixels above baseline, defined as the mean height over the 0.5 s epoch preceding photostimulus onset. All latency measurements reported in the manuscript are based on automated reflectance-based measurements unless otherwise indicated.

#### High-speed video

High-speed video was collected with a Chronos 1.4 camera (Krontech) using a Computar 12.5–75 mm f/1.2 lens sampling at 1000 fps. To synchronize video with stimulation, the camera was triggered with digital pulses sent from the DAQ. Videos were compressed using H.264. Video was analyzed using DeepLabCut^37^ to label the hind paw in sample frames and train a deep neural network to recognize the paw. This returned paw trajectories which were analyzed using custom code written in Python.

#### Pose estimation

DeepLabCut-Live was used to track mouse pose from substage video. While we stimulated only the left hindpaw, networks were trained to recognize the snout, front paws, hind paws, and tail base. The extra keypoints were intended to force the network to assume weights that would represent orientation well, and distinguish between the left and right paws. To train the neural network, we collected one video with 9 mice on the photostimulator platform and panned the camera around under the mice using the linear actuators, collecting 9 min of video. 500 frames were labeled and 95% were used for training a ResNet-50-based neural network with default parameters for 200,000 iterations. We validated on one shuffle and found a test error of 17.41 pixels (px) and train error of 2.62 px. These error values represent multiple keypoints; test error specifically related to the target hind paw is much lower (3.33 px). The image size was 640x480. Importantly, hind paws were not labeled when the paws were turned in a guarding position. This meant that the paws would not be recognized unless placed flat on the platform, and that stimulation would only occur when the paws were correctly oriented. Training was done on a 32 GB NVDIA Tesla V100 GPU, while live inference for aiming was done on a 3 GB NVIDIA Quadro K4000 or NVIDIA GeForce RTX 4070 Ti (see below).

Different networks were required for different applications. To analyze paw withdrawal height, a separate neural network was trained using high-speed video of the mice in profile. DeepLabCut was used with the same parameters as above, training on 580 frames of high-speed video with a 1008x500 resolution. Test error = 5.05 px; train error = 2.32 px. For automated mechanical stimulation, another neural network was trained that could recognize the mouse on a metal grate. Again, the same parameters were used for DeepLabCut, but training on 100 frames with a 1280x800 resolution. Test error = 19.15 px; train error = 1.82 px. To validate photostimulus reliability with automated aiming, a network was trained to recognize a paw-shaped cut out covering a photodiode (see above). The same parameters were used as mentioned above, training on 200 frames with a 640x480 resolution, while labeling the center of the paw-shaped cutout. Test error = 2.43 px; train error = 2.1 px.

#### Automated aiming

The substage camera was aligned with the linear actuators such that movements in the x- and y-directions on video could be independently controlled by x- and y-linear actuators, respectively. The camera was also positioned such that the center of the frame was aligned with the photostimulation zone. x- and y-error signals were then calculated by taking distances from the DeepLabCut-live-based pose estimates for the target paw to the center of the frame (see Figure 7B for example frames). Inference for one frame on an NVIDIA GeForce RTX 4070 Ti could be completed in <20 ms, which is less than the duration of each frame for standard-rate video (1/30 fps = 33 ms). The x- and y-linear actuators were then independently driven with signals that were proportional to the error signals. Importantly, proportionality of the actuator speed to the error signal reduced translation speed as the target was approached, preventing overshoot. Once the target was centered in the frame within a tolerance of 3 px, a timer begins for a user-defined period (2 s for the data shown in Data S1) before stimulation is initiated. This delay assures that the mouse is immobile when initiating stimulation. If the target paw moves during the pre-stimulus interval, aiming is re-initiated and, once aligned, the timer is restarted. A separate timer can be set to stop this sequence and move to the next mouse when a certain mouse is too active to stimulate reliably.

#### Behavior extraction

Substage video was saved and compressed using H.264. DeepLabCut was used to identify the nose, left fore paw, right fore paw, left hind paw, right hind paw, and tail base. These key points were then fed into the VAME framework^64^ using default parameters and 10 clusters to extract complex behaviors.

#### Capsaicin and heat hypersensitivity

A 0.5% w/v solution of capsaicin in mineral oil was prepared. Mice were lightly anesthetized using isoflurane and 5 μL of capsaicin was injected into the left hind paw. Mice were allowed to recover for 15 min from anesthesia before thermal sensitivity was re-assessed.

### Quantification and statistical analysis

Statistical testing was performed using Python 3.8 using SciPy 1.9.3,^74^ Statsmodels 0.13.2,^78^ or with SigmaPlot v11. T-tests were used to compare means, Kolmogorov-Smirnov tests were used to compare distributions, and chi-square tests were used to test differences in frequency of observed behaviors. Visualizations were made with Matplotlib,^76^ Seaborn,^79^ and SigmaPlot.

## Supplemental information

Document S1. Figures S1–S4

Data S1. Sample spreadsheet of fully automated tests with linked videos, related to Figure 7

Table S1. Complete list of components, including sources and part numbers, related to Figures 1 and S1

Document S2. Article plus supplemental information
