# Supplementary material for: Reproducible and fully automated testing of nocifensive behavior in mice
Source: Cell Rep Methods. 2023 Nov 21;3(12):100650. doi: 10.1016/j.crmeth.2023.100650 (PMC10783627; doi:10.1016/j.crmeth.2023.100650)
Supplement: Document S1. Figures S1–S4 [file mmc1.pdf]

Cell Reports Methods, Volume *3*

Supplemental information

Reproducible and fully automated testing

# of nocifensive behavior in mice

Christopher Dedek, Mehdi A. Azadgoleh, and Steven A. Prescott

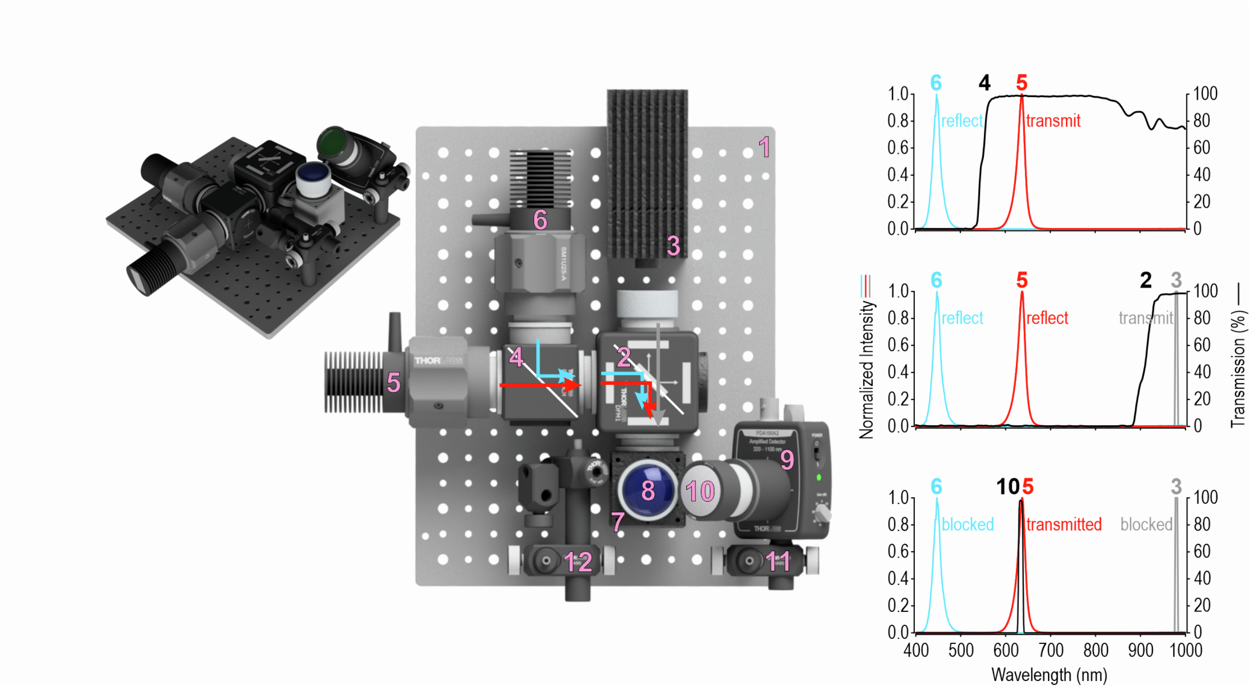

Figure S1. Stimulator components, related to Figure 1.

Key components of the stimulator are numbered 1-12, with details provided in the corresponding entries in Table S1. Emission spectra for the light sources (3, 5, 6) are shown relative to the transmission properties of the dichroic mirrors (2, 4) and notch filter (10) to appreciate how lights sources are combined, and what light is measured by the photodetector (9). All curves except for IR laser are based on data provided by Thorlabs. IR laser is reported to produce 980±5 nm light.

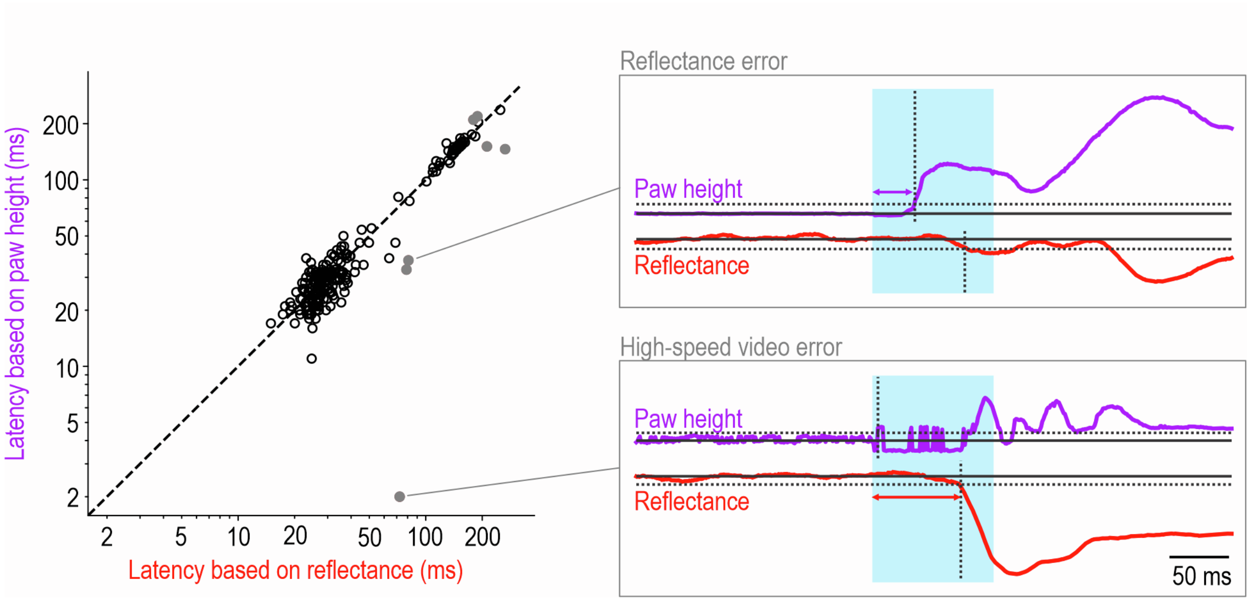

Figure S2. Errors in withdrawal latency measurement, related to Figure 3.

Data are plotted like in Figure 3B but errors trials are now included as grey dots. Of the seven error trials identified through visual inspection of all trials, automated determination of paw position from high-speed video was corrupted by the blue light during photostimulation in 3 trials (top) and the reflectance signal did not immediately change upon paw withdrawal in the other 4 trials (bottom). The false negative rate for the reflectance signal is thus <2% and we did not identify any false positives (i.e. changes in reflectance in the absence of paw movement).

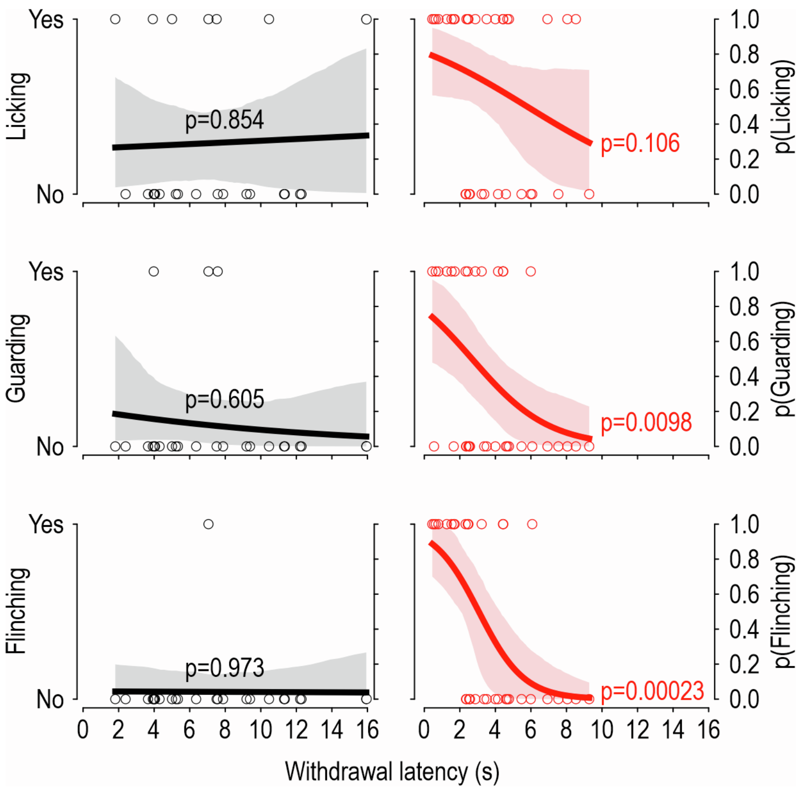

Figure S3. Correlations between the non-reflexive behaviors and latency of the preceding withdrawal, related to Figure 6.

The analysis explained in Figure 6B, which focused on guarding, is extended to licking and flinching using the same trials (*n*=24 at baseline and 34 after capsaicin, from 5 mice). *P* values for logistic regression are indicated on the graphs.

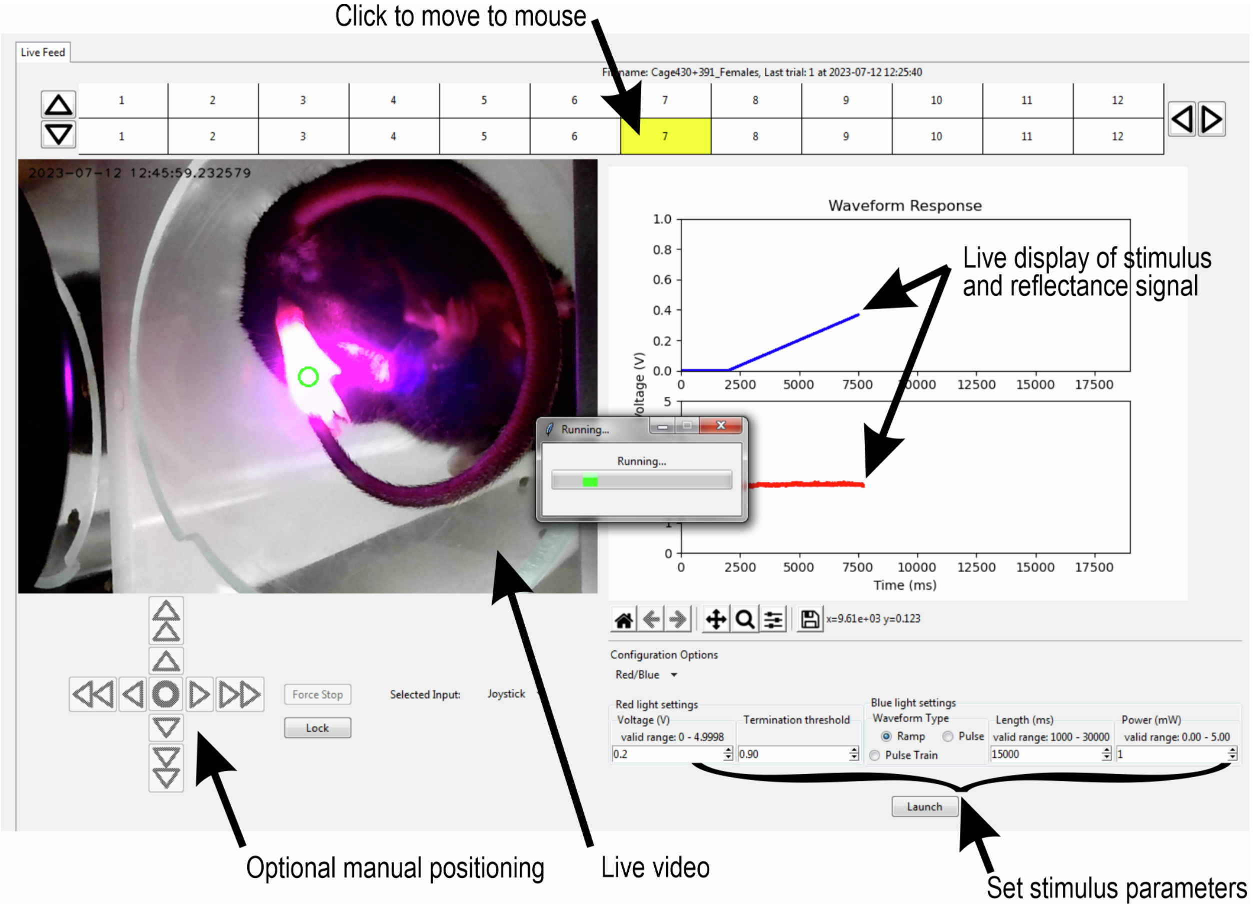

Figure S4. Enlarged view of graphical user interface (GUI), related to Figure 7.

User can toggle between automated and joystick-controlled aiming (bottom left). Top strip identifies which mouse is being tested. Up to 24 mice can be tested, based on two rows of 12 positions. Users can move directly to target mouse by selecting its position. Stimulus parameters are set at the bottom right. All parameters along with date/time of stimulation, mouse identity (position) are automatically saved as metadata to the spreadsheet after each trial (see Fig. 7E and Data S1). User sees a live video of the mouse and a live display of the stimulus intensity and reflectance signal, all of which are recorded to files linked to from the spreadsheet. The withdrawal latency is automatically measured from the reflectance signal and saved to the spreadsheet .
