# Supplementary figures and images for: Reproducible and fully automated testing of nocifensive behavior in mice
Source: Cell Rep Methods. 2023 Nov 21;3(12):100650. doi: 10.1016/j.crmeth.2023.100650 (PMC10783627; doi:10.1016/j.crmeth.2023.100650)

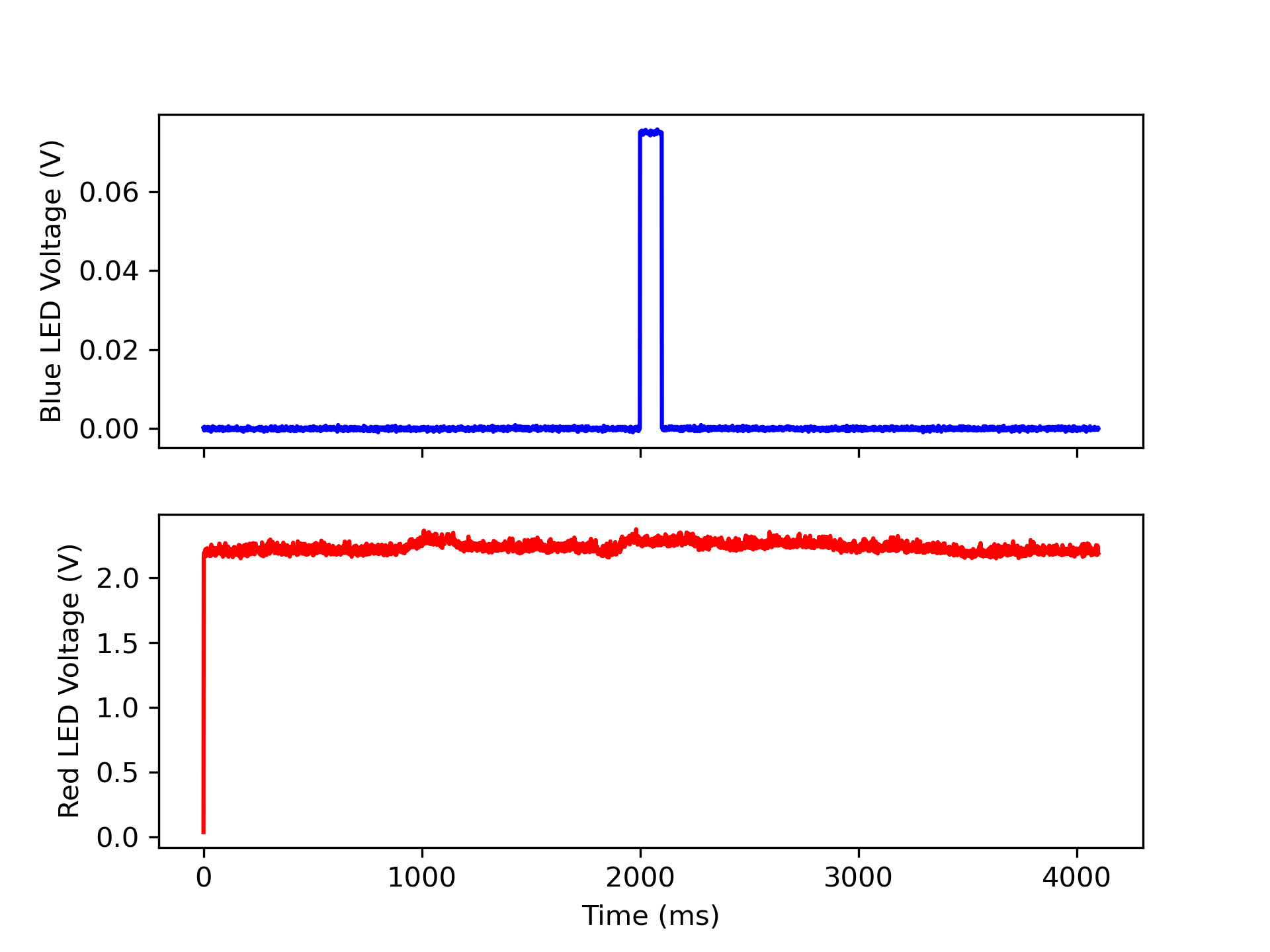

Supplement: Data S1. Sample spreadsheet of fully automated tests with linked videos, related to Figure 7 [file mmc2.zip › TrpV1-ChR2_Example/Cage43_plots/mouseF11.png]

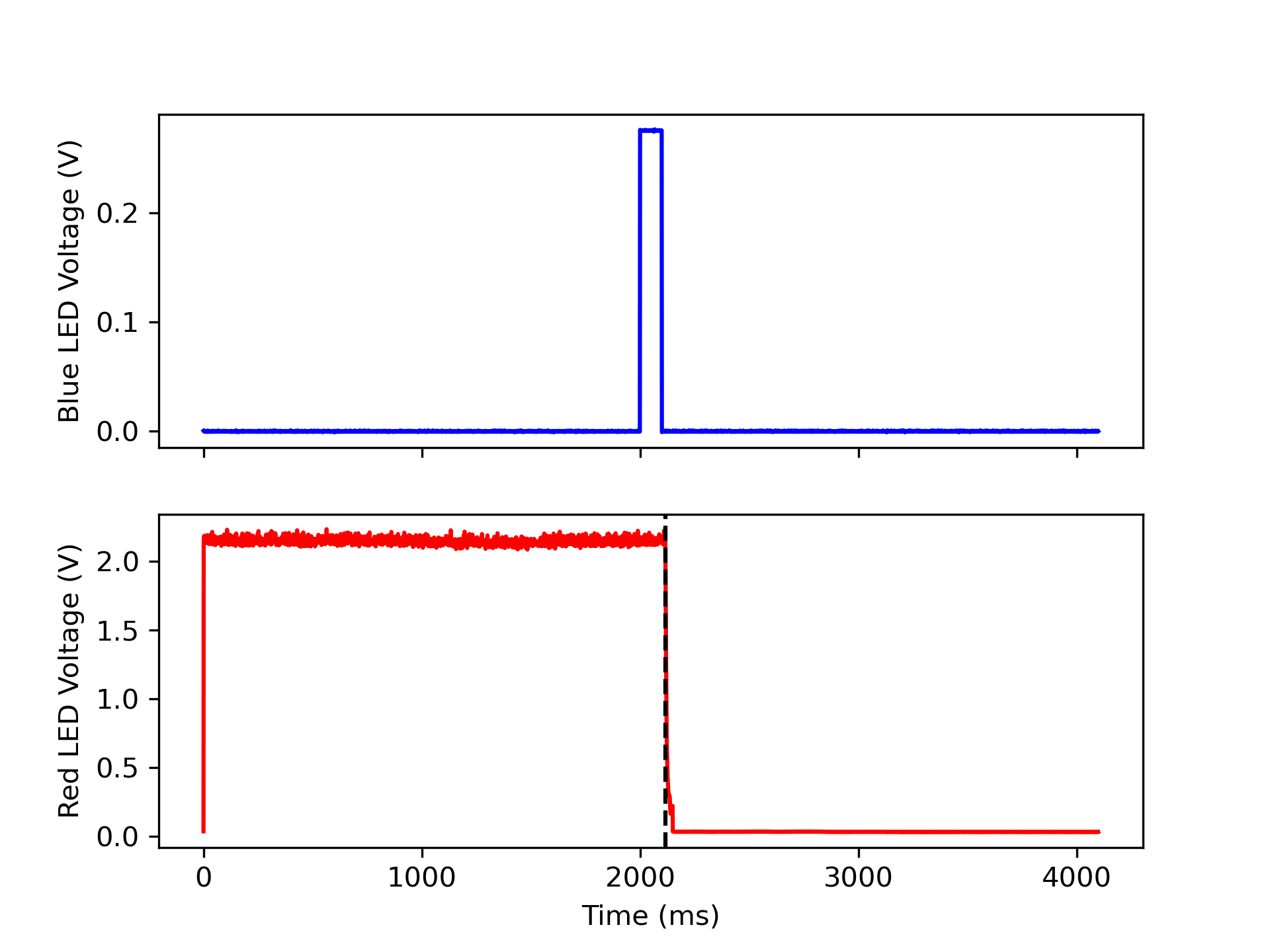

Supplement: Data S1. Sample spreadsheet of fully automated tests with linked videos, related to Figure 7 [file mmc2.zip › TrpV1-ChR2_Example/Cage43_plots/mouseF12.png]

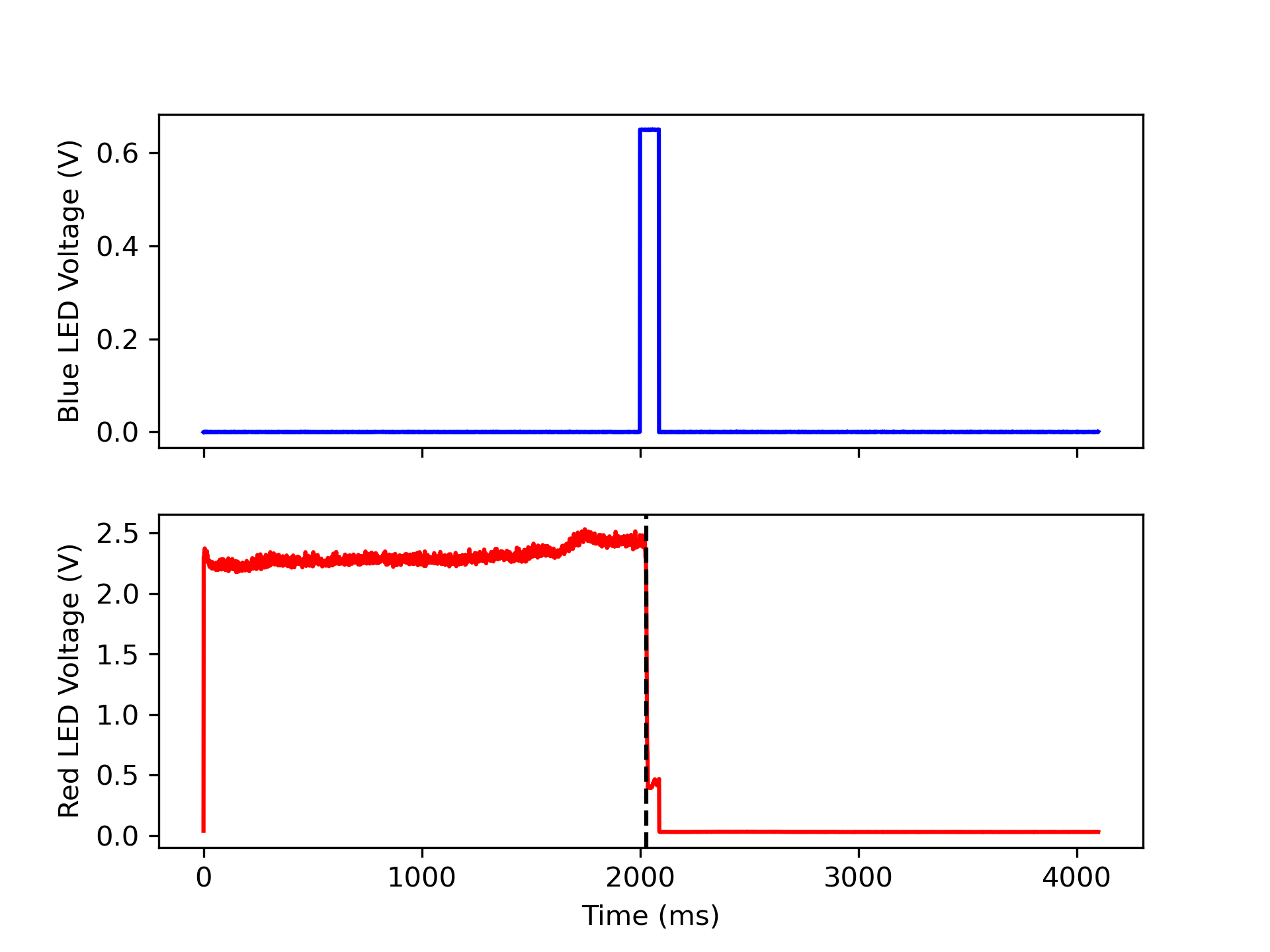

Supplement: Data S1. Sample spreadsheet of fully automated tests with linked videos, related to Figure 7 [file mmc2.zip › TrpV1-ChR2_Example/Cage43_plots/mouseF13.png]

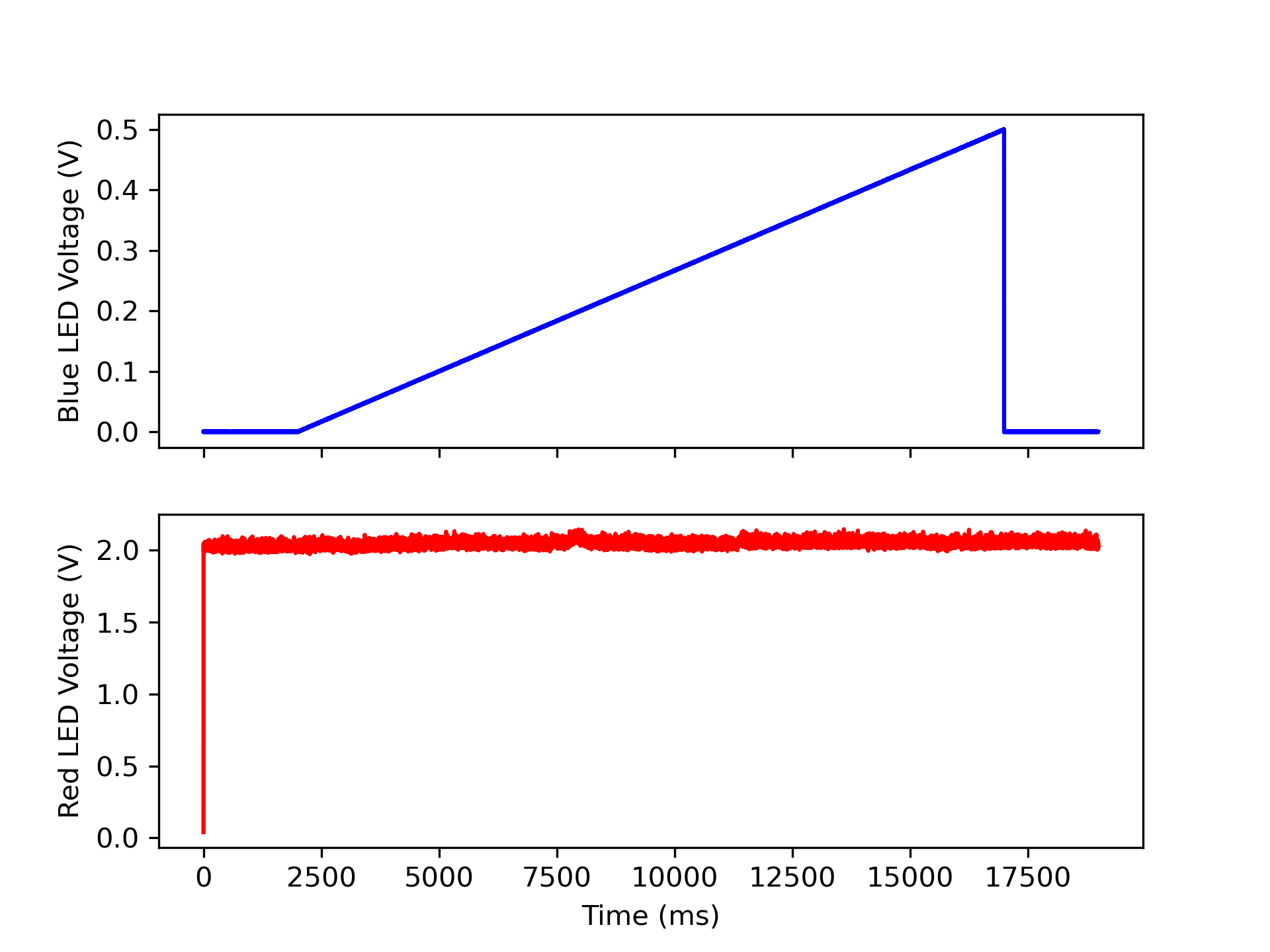

Supplement: Data S1. Sample spreadsheet of fully automated tests with linked videos, related to Figure 7 [file mmc2.zip › TrpV1-ChR2_Example/Cage43_plots/mouseF14.png]

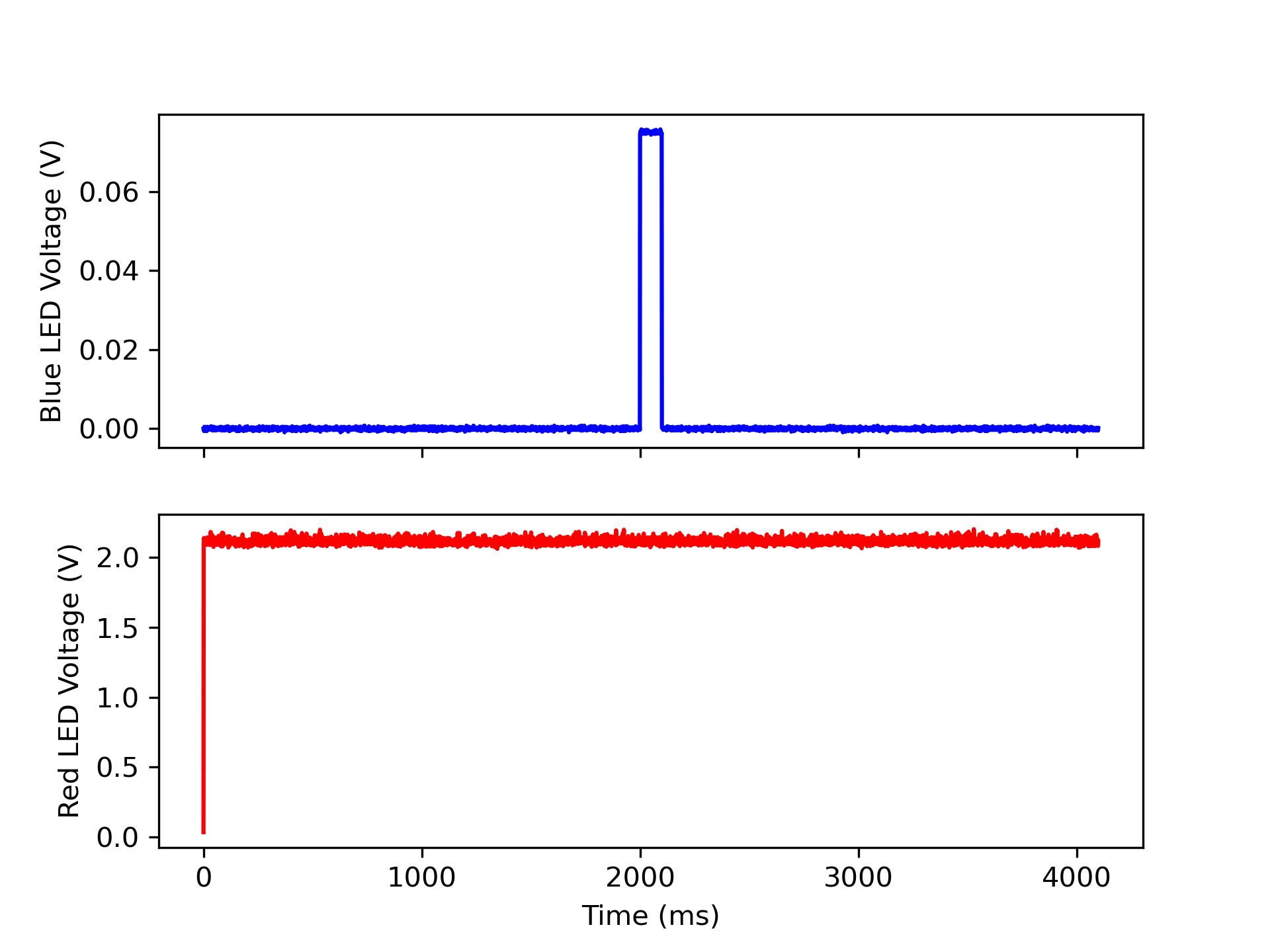

Supplement: Data S1. Sample spreadsheet of fully automated tests with linked videos, related to Figure 7 [file mmc2.zip › TrpV1-ChR2_Example/Cage43_plots/mouseF21.png]

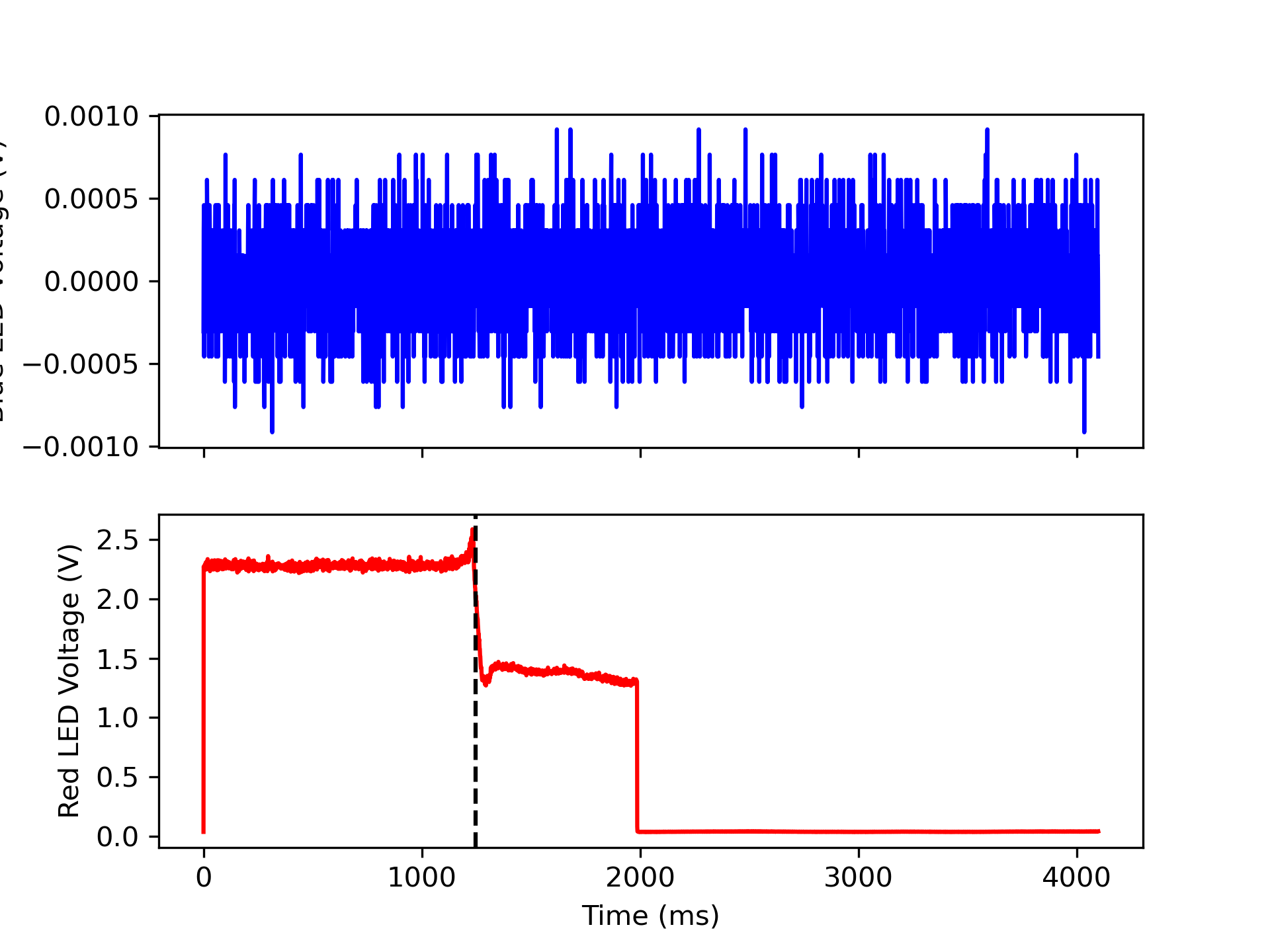

Supplement: Data S1. Sample spreadsheet of fully automated tests with linked videos, related to Figure 7 [file mmc2.zip › TrpV1-ChR2_Example/Cage43_plots/mouseF22.png]

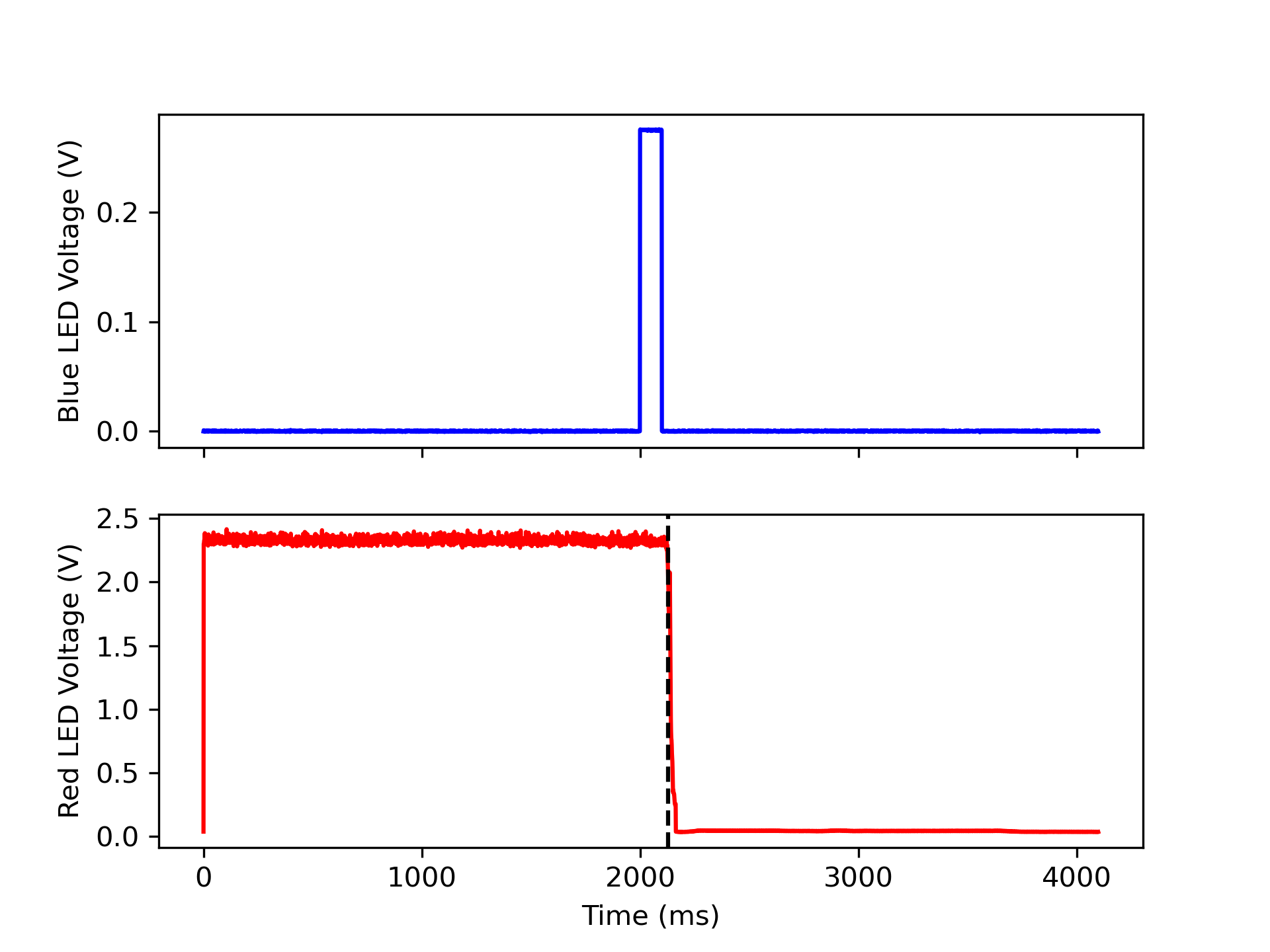

Supplement: Data S1. Sample spreadsheet of fully automated tests with linked videos, related to Figure 7 [file mmc2.zip › TrpV1-ChR2_Example/Cage43_plots/mouseF23.png]

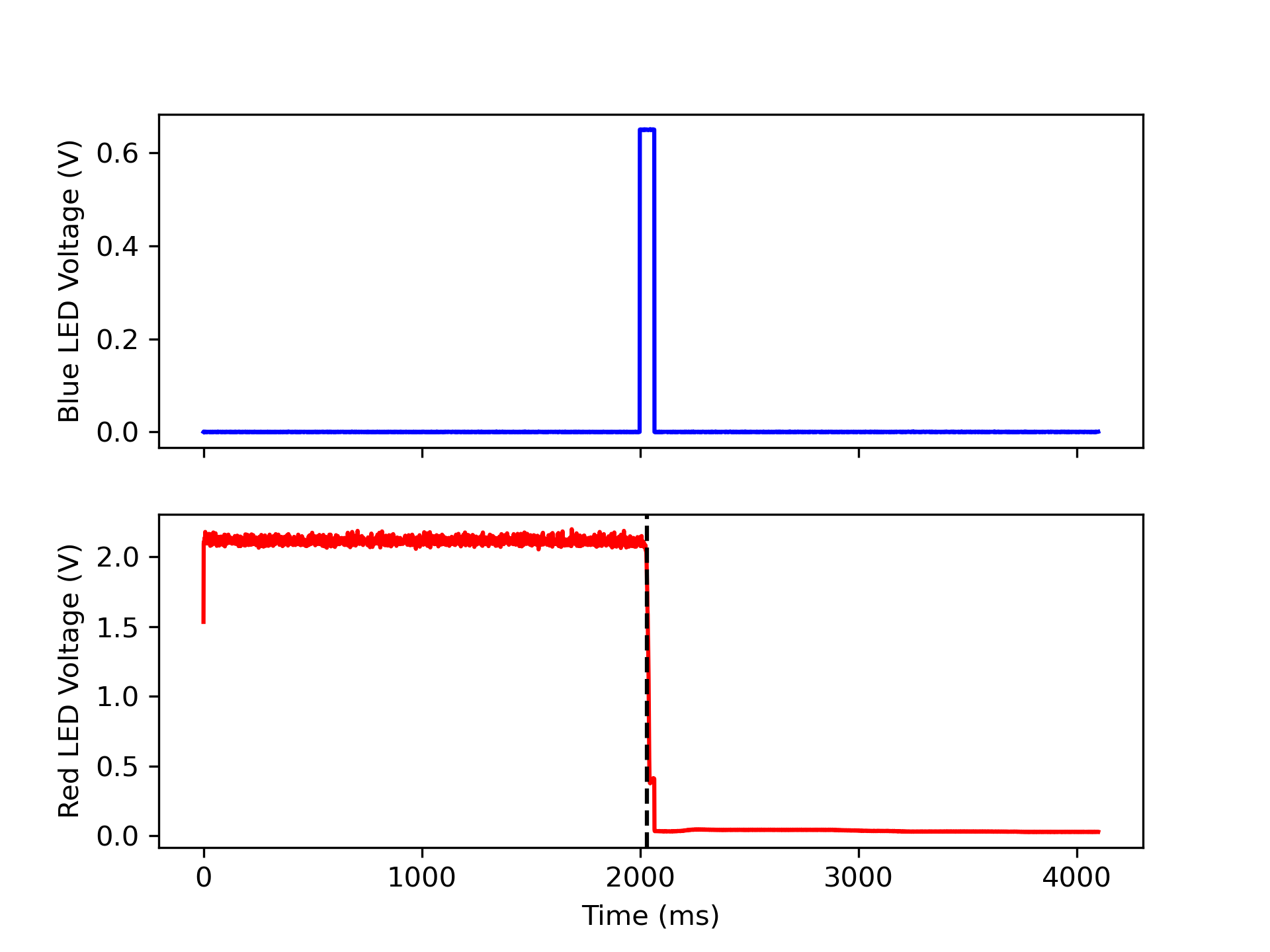

Supplement: Data S1. Sample spreadsheet of fully automated tests with linked videos, related to Figure 7 [file mmc2.zip › TrpV1-ChR2_Example/Cage43_plots/mouseF24.png]

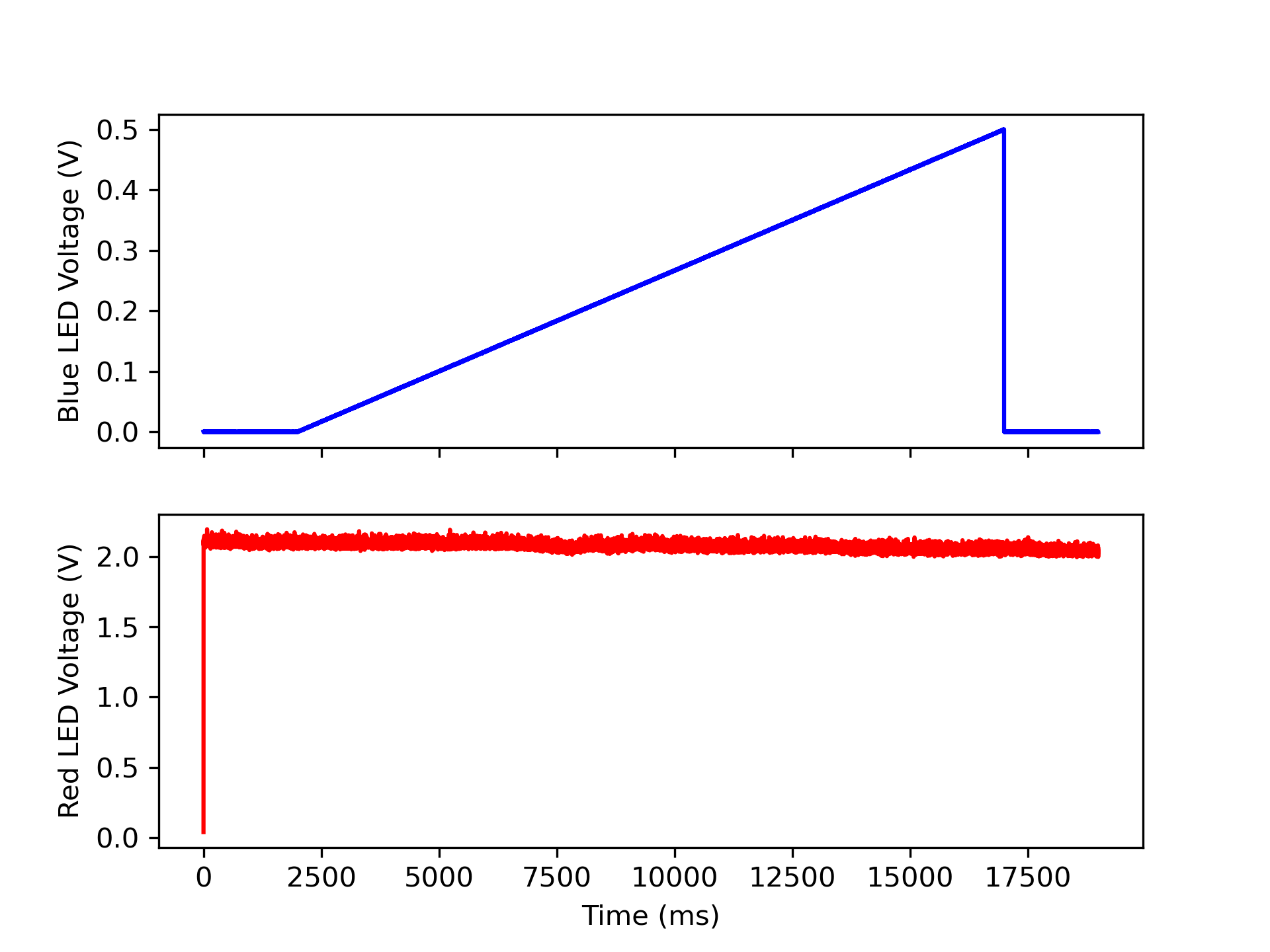

Supplement: Data S1. Sample spreadsheet of fully automated tests with linked videos, related to Figure 7 [file mmc2.zip › TrpV1-ChR2_Example/Cage43_plots/mouseF25.png]

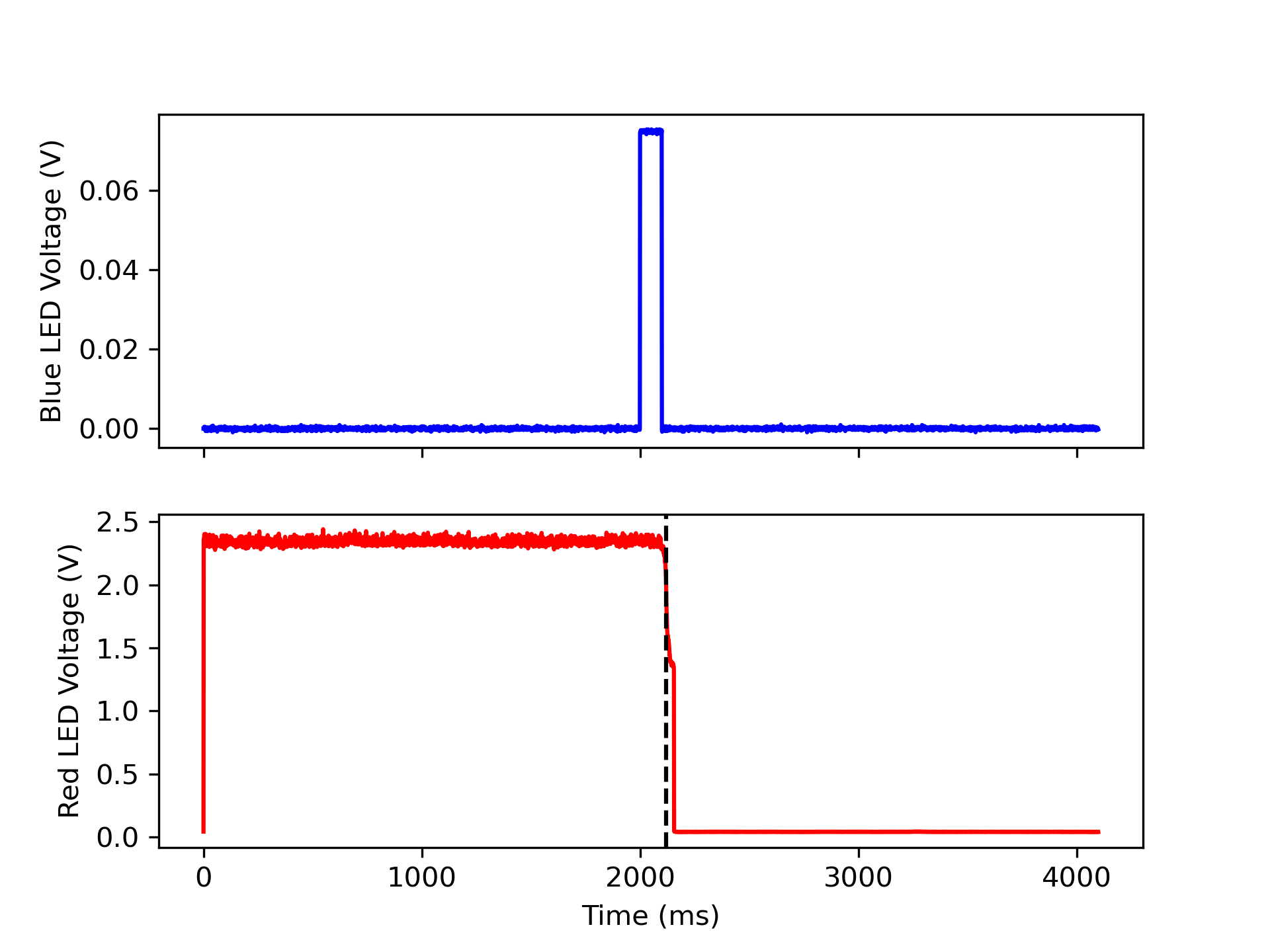

Supplement: Data S1. Sample spreadsheet of fully automated tests with linked videos, related to Figure 7 [file mmc2.zip › TrpV1-ChR2_Example/Cage43_plots/mouseF31.png]

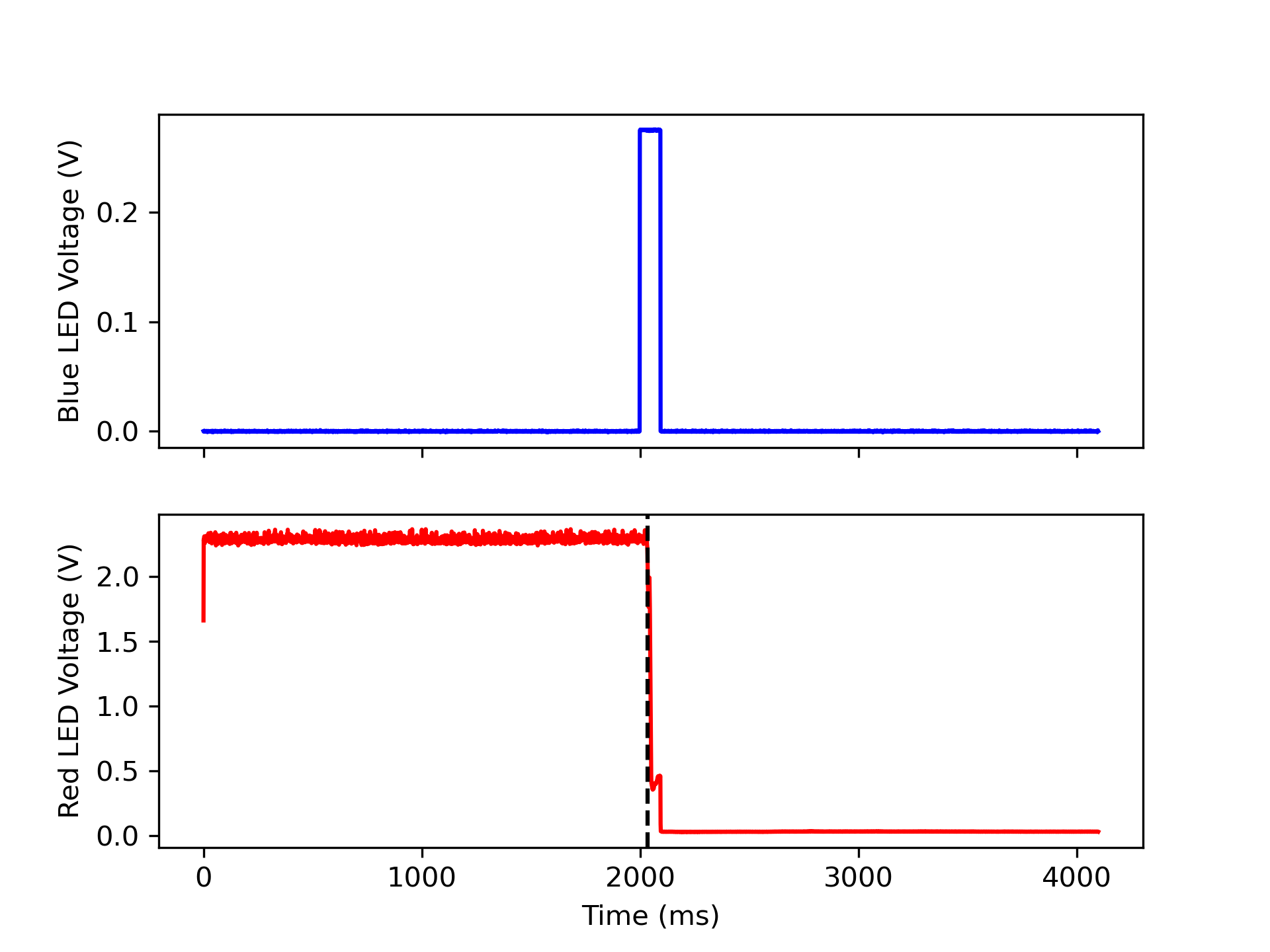

Supplement: Data S1. Sample spreadsheet of fully automated tests with linked videos, related to Figure 7 [file mmc2.zip › TrpV1-ChR2_Example/Cage43_plots/mouseF32.png]

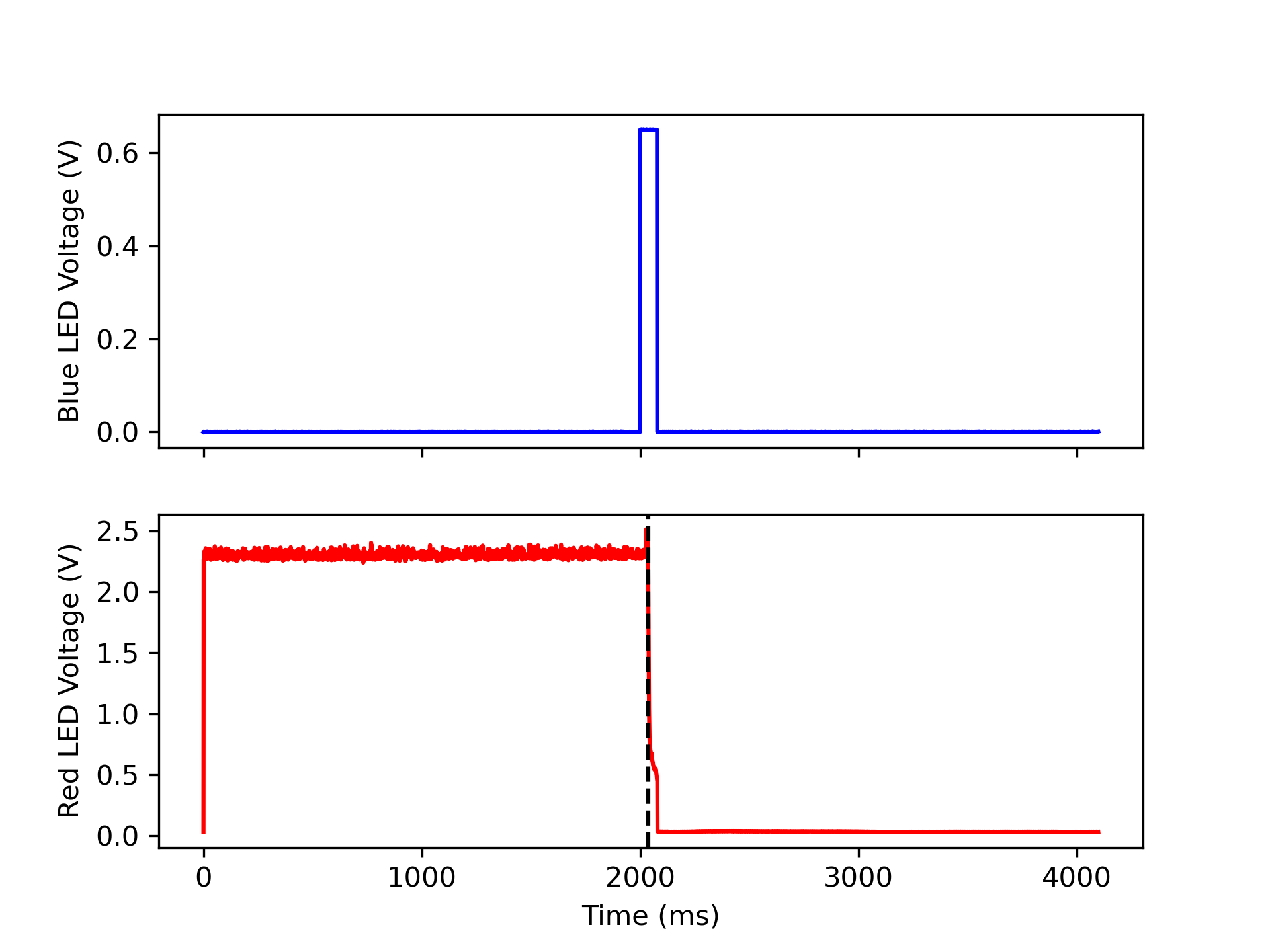

Supplement: Data S1. Sample spreadsheet of fully automated tests with linked videos, related to Figure 7 [file mmc2.zip › TrpV1-ChR2_Example/Cage43_plots/mouseF33.png]

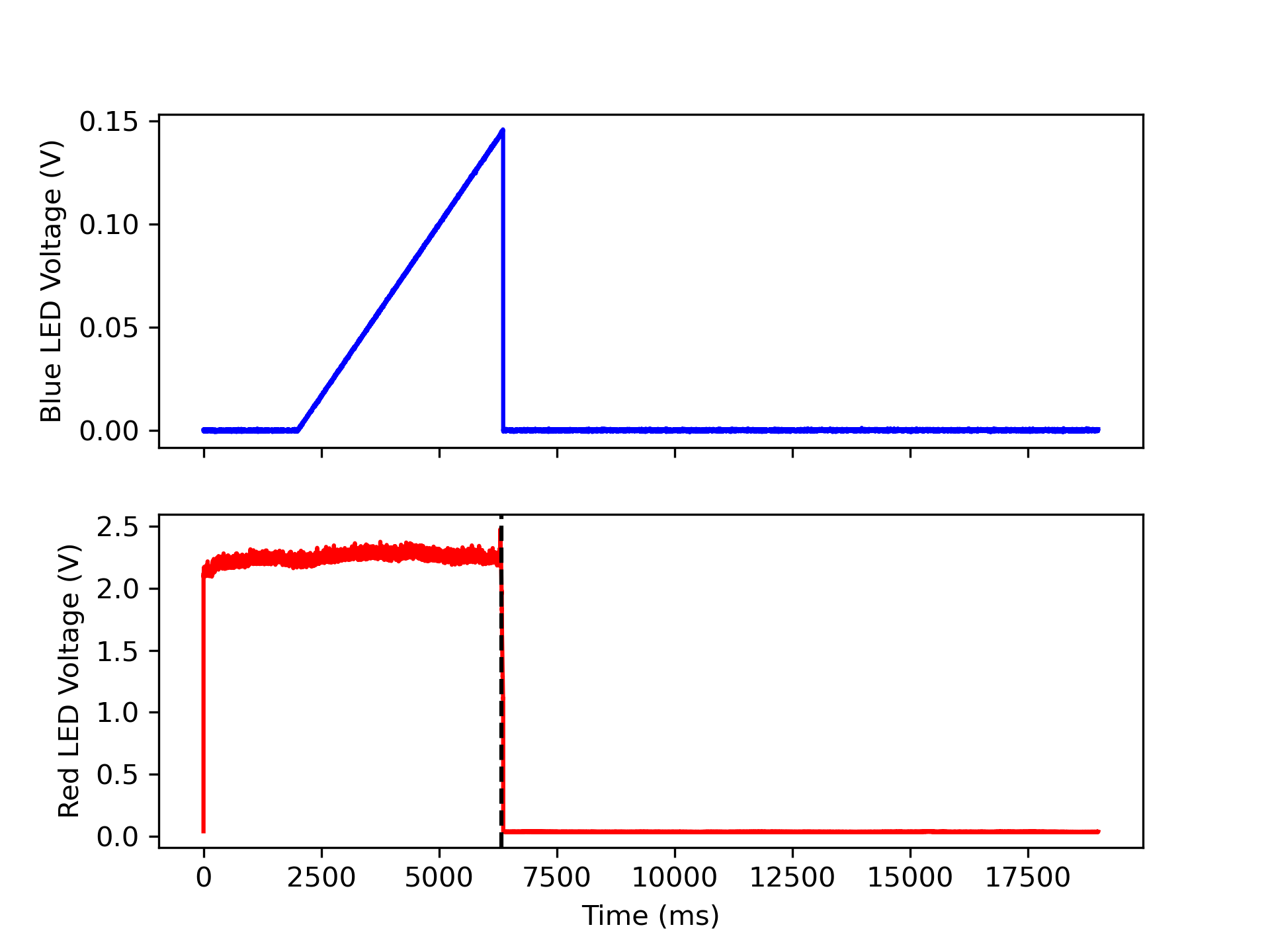

Supplement: Data S1. Sample spreadsheet of fully automated tests with linked videos, related to Figure 7 [file mmc2.zip › TrpV1-ChR2_Example/Cage43_plots/mouseF34.png]
